# Highly Oxidized Ecdysteroids from a Commercial *Cyanotis arachnoidea* Root Extract as Potent Blood–Brain Barrier Protective Agents

**DOI:** 10.1021/acs.jnatprod.2c00948

**Published:** 2023-02-24

**Authors:** Gábor Tóth, Ana R. Santa-Maria, Ibolya Herke, Tamás Gáti, Daniel Galvis-Montes, Fruzsina R. Walter, Mária A. Deli, Attila Hunyadi

**Affiliations:** † Department of Inorganic and Analytical Chemistry, NMR Group, Budapest University of Technology and Economics, H-1111 Budapest, Hungary; ‡ Institute of Biophysics, Biological Research Centre, Szeged H-6726, Hungary; § Wyss Institute for Biologically Inspired Engineering at Harvard University, Boston, Massachusetts 02115, United States; ⊥ Servier Research Institute of Medicinal Chemistry (SRIMC), H-1031 Budapest, Hungary; ^¶^Institute of Pharmacognosy, and ^∥^Interdisciplinary Centre of Natural Products, University of Szeged, H-6720 Szeged, Hungary

## Abstract

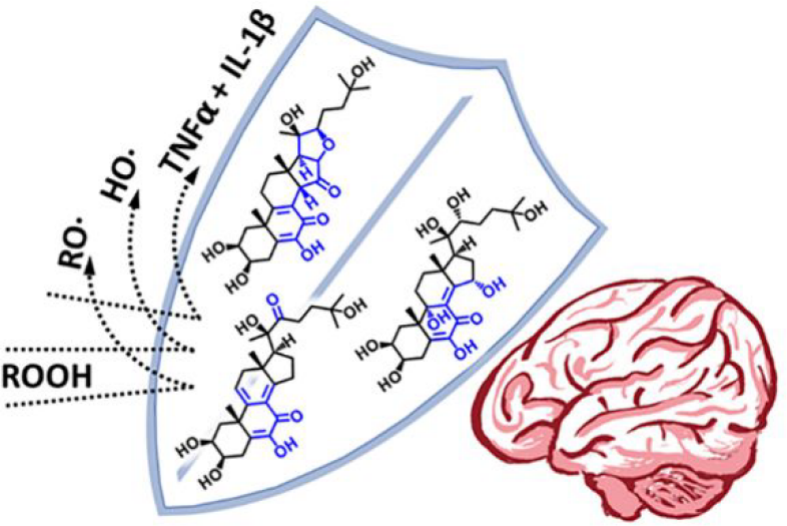

Ecdysteroid-containing herbal extracts, commonly prepared from the roots of *Cyanotis arachnoidea*, are marketed worldwide as a “green” anabolic food supplement. Herein are reported the isolation and complete ^1^H and ^13^C NMR signal assignments of three new minor ecdysteroids (compounds **2**–**4**) from this extract. Compound **4** was identified as a possible artifact that gradually forms through the autoxidation of calonysterone. The compounds tested demonstrated a significant protective effect on the blood–brain barrier endothelial cells against oxidative stress or inflammation at a concentration of 1 μM. Based on these results, minor ecdysteroids present in food supplements may offer health benefits in various neurodegenerative disease states.

Phytoecdysteroids represent an abundant and widespread group of natural products that includes the insect-molting hormone 20-hydroxyecdysone (20E) and its structurally diverse analogues.^1^ While 20E is toxic to insects and acts as a plant chemical defense agent against nonadapted pests,^2^ it is acknowledged widely for its beneficial pharmacological effects in mammals including humans. Without any detectable hormonal activity,^3^ 20E has broad pharmacological effects including adaptogen, general strengthening, cytoprotective, and anabolic activities in vertebrates.^4−6^ It was revealed recently that its diverse bioactivities are mediated via the activation of the protective arm of the renin–angiotensin system through the Mas1 receptor.^7^ Consistent with its cytoprotective action, 20E was also reported to ameliorate oxidative stress-induced neuronal injury in vitro and to act as a neuroprotective agent in vivo in an ischemic reperfusion model.^8^

Ecdysteroid-containing herbal food supplements are available worldwide for human consumption at a relatively inexpensive price.^9^ These food supplements typically originate from the roots of *Cyanotis arachnoidea* C. B. Clarke (Commeliniaceae). In our previous work, it was demonstrated that commercial extracts of this herbal drug contain rare minor ecdysteroids and that such compounds have the potential for the development of new compounds for biological pest management.^10^

Damage to the blood–brain barrier (BBB) is a key mechanism in the pathogenesis of many acute (e.g., stroke, traumatic brain injury) and chronic central nervous system (CNS) pathologies (e.g., Alzheimer’s, Parkinson’s, and Huntington’s disease).^11−13^ Thus far, the possible BBB-protective activity of ecdysteroids has not been studied. Furthermore, while the cytoprotective effect of 20E in mammals has been relatively well described, little is known about the pharmacology of minor ecdysteroids. Therefore, as a follow-up to our recent report,^10^ in this current study it was the aim to further evaluate the chemical value of the commercially available *Cyanotis* extract and the pharmacological value of the isolated ecdysteroids with regard to their potential cytoprotective effects on the BBB.

The dried extract of *C. arachnoidea* roots was further extracted with methanol and subjected to a stepwise chromatographic purification as described in detail in the Supporting Information. Three minor ecdysteroids were obtained. The structure of the isolated compounds was elucidated based on their molecular formulas, which were obtained by high-resolution mass spectrometry (HRMS) and on detailed nuclear magnetic resonance (NMR) studies. A preliminary assessment of the NMR spectra revealed that the currently isolated compounds **2**–**4** shared structural similarities with 14β,15-dihydrocalonysterone (**1**, C_27_H_42_O_7_) that was recently reported by our group;^10^ therefore, the structure elucidation is described in relation to this compound.

The ^1^H and ^13^C NMR chemical shifts of compounds **1**–**4** are presented in Table 1. The HRMS of the new compounds (**2**–**4**) are presented in Figures S1, S8, and S16 (Supporting Information), and their characteristic one-dimensional ^1^H, selective-TOCSY, selective-ROESY, ^13^C-DEPTQ, and two-dimensional edited heteronuclear single quantum coherence (HSQC) and heteronuclear multiple bond correlation (HMBC) NMR spectra, along with their stereostructures, ^1^H and ^13^C NMR assignments, and characteristic HMBC correlations and steric proximities, are all presented in Figures S2–S7, S9–S15, and S17–S23 (Supporting Information). The structures of compounds **1**–**4** are shown in Figure 1.

**Table 1:** ^1^H and ^13^C NMR Chemical Shifts of Compounds **1**–**4** in DMSO-*d*_6_

	**1**^a^		**2**^a^		**3**^a^		**4**^b^	
no.	^1^H	^13^C	^1^H	^13^C	^1^H	^13^C	^1^H	^13^C
1β	2.17	40.3	2.17	41.1	1.64	41.5	1.64	34.8
α	1.14		1.13		2.17		2.17	
2α	3.80	68.0	3.81	68.0	3.91	68.1	3.91	68.6
3α	3.31	71.7	3.33	71.9	3.39	72.0	3.39	69.3
4β	2.35	26.8	2.36	26.8	2.30	26.8	2.30	26.8
α	2.89		2.93		2.80		2.80	
5		133.2		133.7		132.8		136.9
6		142.6		142.6		142.6		142.8
7		180.2		179.1		179.4		185.2
8		131.5		124.8		122.7		129.9
9		161.7		161.3		163.9		73.7
10		40.5		40.76		40.8		42.2
11β	2.32	22.4	∼2.34	21.4	2.12	125.7	2.12	27.0
α	2.22		∼2.34		1.54		1.54	
12β	1.72	35.0	1.56	34.0	1.96	35.8	1.96	31.5
α	1.37		1.07		1.62		1.62	
13		40.4		35.5		45.9		46.7
14β	2.43	46.4	4.05	50.7		141.0		169.5
15β	2.08	30.2		212.5	4.58	23.7	4.58	68.9
α	0.92							
16β	1.70	25.2		80.1	2.21	31.7	2.21	31.7
α	1.50		4.10		1.57		1.57	
17α	1.68	50.8	2.34	57.6	1.93	56.1	1.93	51.5
18	1.10	23.8	1.22	17.1	1.02	17.3	1.02	18.8
19	1.36	26.5	1.35	27.0	1.20	27.1	1.20	27.3
20		75.6		78.8		79.8		75.2
21	1.13	19.9	1.31	23.6	1.12	24.6	1.12	20.9
22	3.20	76.4	3.33	89.9	3.23	217.3	3.23	76.2
23	1.42	26.0	1.51	22.2	1.46	32.1	1.46	26.1
	1.13		1.43		1.12		1.12	
24	1.64	41.3	1.57	40.8	1.63	37.0	1.63	41.3
	1.13		1.31		1.26		1.26	
25		68.7		68.7		68.1		68.8
26	1.03	29.0	1.07	29.1	1.04	29.3	1.04	29.2
27	1.05	29.9	1.08	29.7	1.06	29.34	1.06	29.9
HO-2	4.57		4.59		4.62		4.43	
HO-3	4.87		4.93		4.92		4.77	
HO-6	8.02		8.15		8.07		7.89	
HO-9							4.94	
HO-15							5.39	
HO-20	3.61		4.73		5.02		3.76	
HO-22	4.33						4.45	
HO-25	4.07		4.13		4.19		4.15	

a 500/125 MHz.

b 600/150 MHz.

**Figure 1:**
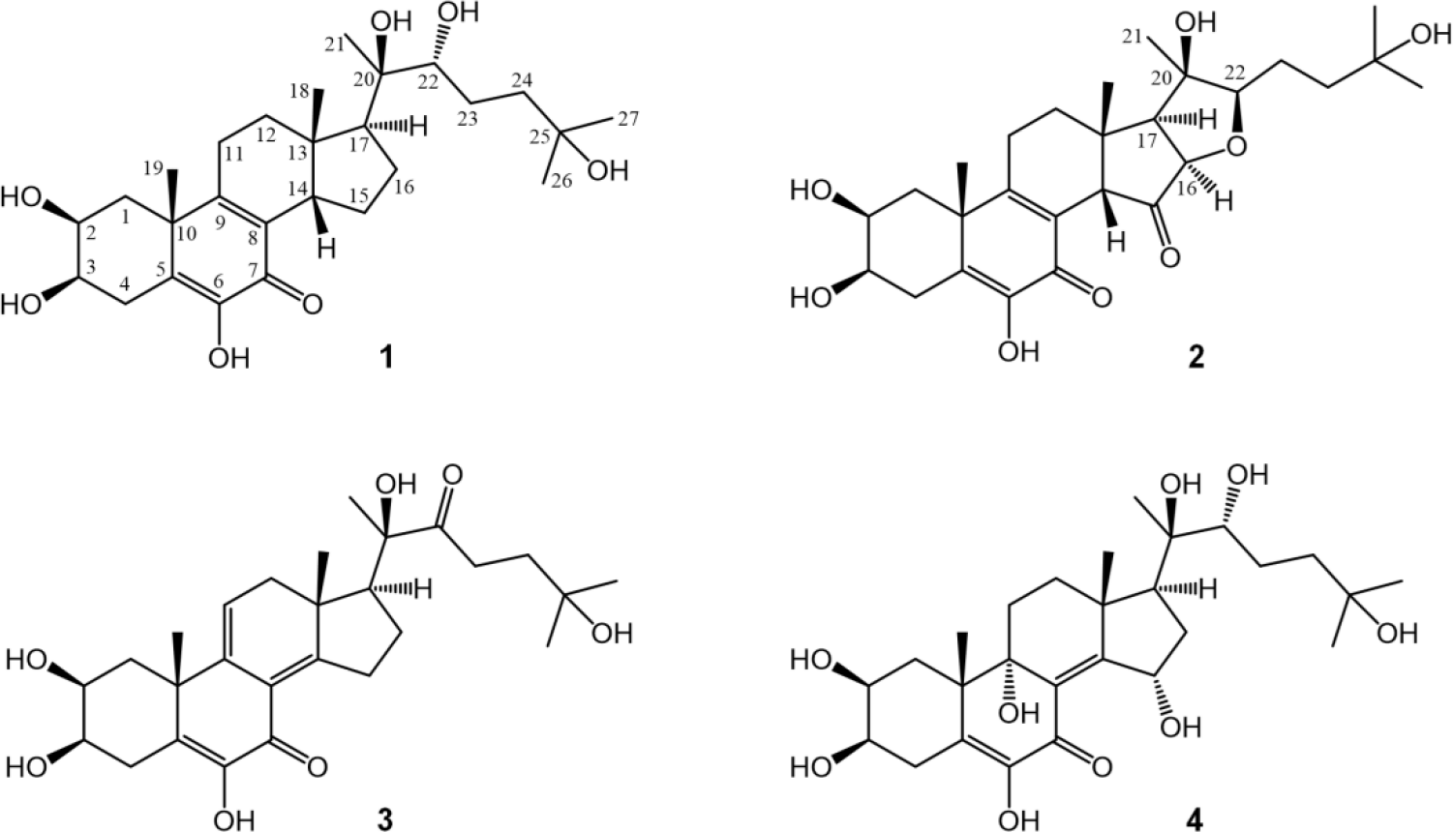
Structures of the new compounds **2**–**4** in comparison with that of the previously reported 14β,15-dihydrocalonysterone (**1**).

For compound **2**, the HRMS data indicated an elemental composition of C_27_H_38_O_8_; i.e., this compound is a C_27_-ecdysteroid with an intact side chain. The molecule consists of one oxygen atom more and four hydrogen atoms less than the reference compound **1**, and the number of its double bond equivalents increased to 9; i.e., it contains five rings and four double bonds. For the structure elucidation and NMR signal assignments, the following NMR spectra were recorded: ^1^H, selective-ROE irradiating CH_3_-19, -18, and -21, selective-ROE irradiating Hα-16, Hβ-14, and Hα-22 and H-3, ^13^C DEPTQ, edited-HSQC, and HMBC (Figures S2–S7, Supporting Information). The ^1^H and ^13^C NMR chemical shifts detected (see Table 1) of the A, B, and C rings of the steroid core were similar to those of compound **1**. The edited HSQC experiment (Figure S6, Supporting Information) revealed a significant difference in the D ring, and the signals of C=O (δ_C-15_ 212.5 ppm) and O–CH units (δ_C-16_ 80.1 ppm, δ_H-16_ 4.10 d *J*_16,17_ = 9.0 Hz) appeared instead of the 15,16 −CH_2_–CH_2_– moiety of compound **1**. In the HMBC spectrum (Figure S7, Supporting Information), the cross-peaks of the H_3_-18 hydrogens were used to assign the δ_C-14_ and δ_C-17_ methines to 50.7 and 57.6 ppm, whereas the corresponding H-14 and H-17 methine hydrogens (4.05 s and 2.34 d, ppm) correlated with the C=O carbon signal at 212.5 ppm. In addition, the NMR signals of the side chain (C-20–C-27) coupled to C-17 were similar between compounds **1** and **2** except for the δ_C-22_ signal at 89.8 ppm because this methine signal in compound **2** showed a paramagnetic shift of 13.5 ppm. This phenomenon indicated that a new ring was formed through a H–C(16)–O–C(22) connection. For the elucidation of the stereochemistry of the H-14, H-16, H-17, and H-22 hydrogens, selective-ROESY experiments were performed. The irradiation of H_3_-18 resulted in an NOE interaction with Hβ-14, whereas the irradiation of H_3_-21 proved the steric proximities and the Hα-17 and Hα-22 orientations. Selective-ROE experiments of Hα-16 and Hα-22 showed their steric proximity and thus their alpha orientation (Figures S3 and S4, Supporting Information).

The HRMS measurement of compound **3** established the molecular formula C_27_H_38_O_7_, i.e., a C_27_-ecdysteroid containing four rings, one HC=C, two C=C double bonds, one C=O, and one additional conjugated C=O group, as indicated by the DEPTQ spectrum (Figure S12, Supporting Information). The structure elucidation and NMR assignments were based on the following spectra: ^1^H, selective-TOCSY on H-2, Hα-4, Hα-17, and Hα-16, selective-ROE irradiating CH_3_-19, -21, and -18, ^13^C DEPTQ, HSQC, edited-HSQC, and HMBC (Figures S9–S15, Supporting Information). The selective-TOCSY experiments allowed the selective detection of the hydrogen signals of the A and D rings. Selective-ROE experiments on H_3_-19, H_3_-21, and H_3_-18 differentiated between the α and β orientation of each hydrogen atom located in steric proximity to the irradiated methyl signals. The 1.31/217.3 ppm cross-peak in the HMBC spectrum (Figure S15, Supporting Information) unambiguously revealed the C-22 position of the carbonyl group on the side chain.

For compound **4**, the HRMS data indicated an elemental composition of C_27_H_42_O_9_, i.e., a C_27_-ecdysteroid with seven double bond equivalents. For its structure elucidation and NMR signal assignments, the following spectra were obtained: ^1^H, selective-TOCSY irradiating H-15, H-2, and H-22, selective-ROE irradiating CH_3_-18, -21, and -19, ^13^C DEPTQ, edited-HSQC, and HMBC (Figures S17–S23, Supporting Information). The DEPTQ spectrum of compound **4** exhibited 27 ^13^C NMR signals, indicating the presence of five methyls, seven methylenes, four sp^3^ HC–O groups, one CH methine group, two C=C double bonds, one conjugated C=O (δ 185.2 ppm), and five quaternary sp^3^ carbon atoms. The NMR signals of the side chain (C-20–C-27) coupled to C-17 were similar for compounds **4** and **1** because this structural element is identical for these two molecules. The selective TOCSY (Figure S18, Supporting Information) of H-22 revealed the H-22–H-24 signals, whereas the same experiment on H-2 identified the entire spin system of the A ring, including the HO-2 and HO-3 hydrogen atoms, without any overlap. The assignment of the hydrogen spin system in the D ring was supported by the selective-TOCSY procedure on H-15, and the Hβ-15 configuration was proven through the detected NOE response on it, when irradiating CH_3_-18 hydrogens in the selective-ROE experiment (Figure S19, Supporting Information). The position of the hydrogens on the steroid skeleton was confirmed by ROE experiments. The complete and unambiguous signal assignment of compound **4** was achieved through utilizing the edited HSQC and HMBC data.

Compounds **2**–**4** are highly oxidized new ecdysteroids that express many unusual structural elements. The structure of compound **2** is particularly notable; it has the rare *o*-quinol B ring and 14β-hydrogen, which was recently identified in compound **1**, as well as two new structural elements, i.e., a 15-keto group and a tetrahydrofuran ring formed between C-22 and C-16. The resulting pentacyclic structure represents a new ecdysteroid skeleton. Compound **3** has an unprecedented conjugated double bond system in its B and C rings, and compound **4** is unique with its calonysterone-like B ring accompanied by a 9,15-diol and a Δ^8–14^ olefin. Compound **4** was isolated from a crystallization mother liquid of calonysterone (see the Supporting Information) and was also identified in several previously purified calonysterone-containing fractions. This, together with its oxidized calonysterone-like chemical structure, indicated that compound **4** might be a decomposition product of calonysterone. To evaluate this possibility, an aliquot of calonysterone was dissolved in 50% aqueous methanol and kept on a desk for 20 weeks extended time. The slow, gradual autoxidation of calonysterone was confirmed by repeated analytical HPLC measurements, and compound **4** was identified unambiguously as a single major product by a combination of HPLC-photodiode array (PDA) and thin-layer chromatography followed by spraying with vanillin-sulfuric acid. The time dependency of the oxidation of calonysterone into compound **4** is shown in Figure 2, and HPLC-PDA fingerprint chromatograms taken at 4, 9, 14, 16, and 20 weeks are provided in Figure S24 (Supporting Information).

**Figure 2:**
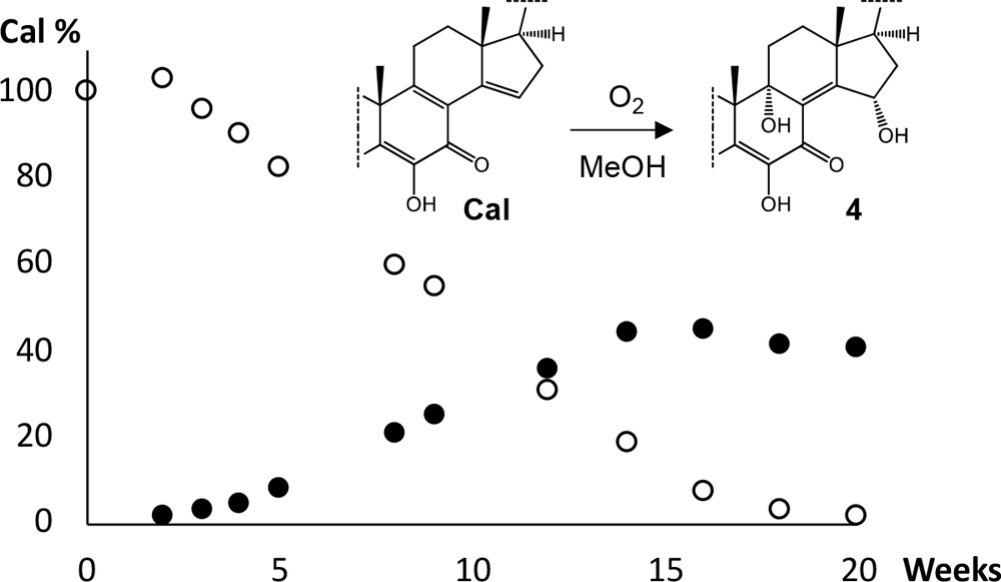
Time-dependent study of the decomposition of a 1 mg/mL 50% methanol solution of calonysterone (Cal; open circles) into compound **4** (filled dots). Amounts are given as relative percentages of the initial amount of calonysterone.

The present results strongly suggest that compound **4** is an artifact in the commercial plant extract, and it was formed through the autoxidation of calonysterone. Due to the industrial origin of the extract that was processed under unknown conditions, in place of a freshly collected plant material sample, no definitive judgment can be made regarding the genuine nature of compounds **2** and **3**. Nevertheless, it must be stressed that these extracts are consumed worldwide by humans as food supplements;^9^ therefore, studying its composition and possibly related implications concerning human health and disease is of high importance. Calonysterone is a major autoxidized product of the abundant 20E, and under appropriate conditions it may be produced from 20E with a high yield.^14^ The present results show that in solution the aerobic oxidation may yield as much as 45% of compound **4**. This has two major implications: (i) these compounds are likely to be present in any 20E-containing food supplement, and (ii) the preparation of compound **4** at an industrial scale is possible.

The effects of compounds **2**–**4** were tested on hCMEC/D3 human brain microvascular endothelial cells for cell barrier integrity and viability, using impedance measurements. The concentration range of 0.01–10 μM of compounds **2**–**4** was evaluated, and no significant change in cellular impedance indicating altered barrier tightness and viability was observed, with two exceptions. First, the 10 μM concentration of compound **2** at the 4 h treatment point significantly decreased cell impedance, whereas no change was observed for compounds **3** and **4**. Second, a significant cell impedance increase was detected for compound **3** at a 1 μM concentration. For the other compounds, a trend of increased cellular impedance was observed (Figure S26, Supporting Information). Based on these results, a 1 μM concentration of the three compounds was selected for further testing.

Oxidative stress promotes disruption of the blood–brain barrier (BBB) by the excess production of reactive oxygen species (ROS) followed by a compromised antioxidant defense. Oxidative damage on the cellular components (proteins, lipids, and DNA), modulation of tight junctions, the activation of matrix metalloproteinases, and the upregulation of inflammatory molecules are consequences of BBB damage caused by ROS.^15^ To evaluate the protective effect of **2**–**4** against damage caused by ROS, cells were treated with *tert*-butyl hydroperoxide (tBHP; 350 μM) alone or in combination with the test compounds. tBHP produces high amounts of ROS that induces cellular damage.^16^ The optimal concentration of tBHP was evaluated in preliminary experiments, and the concentration of 350 μM, which did not decrease the cell index below ∼50%, was selected for the protection assay, with the results shown in Figure 3.

**Figure 3:**
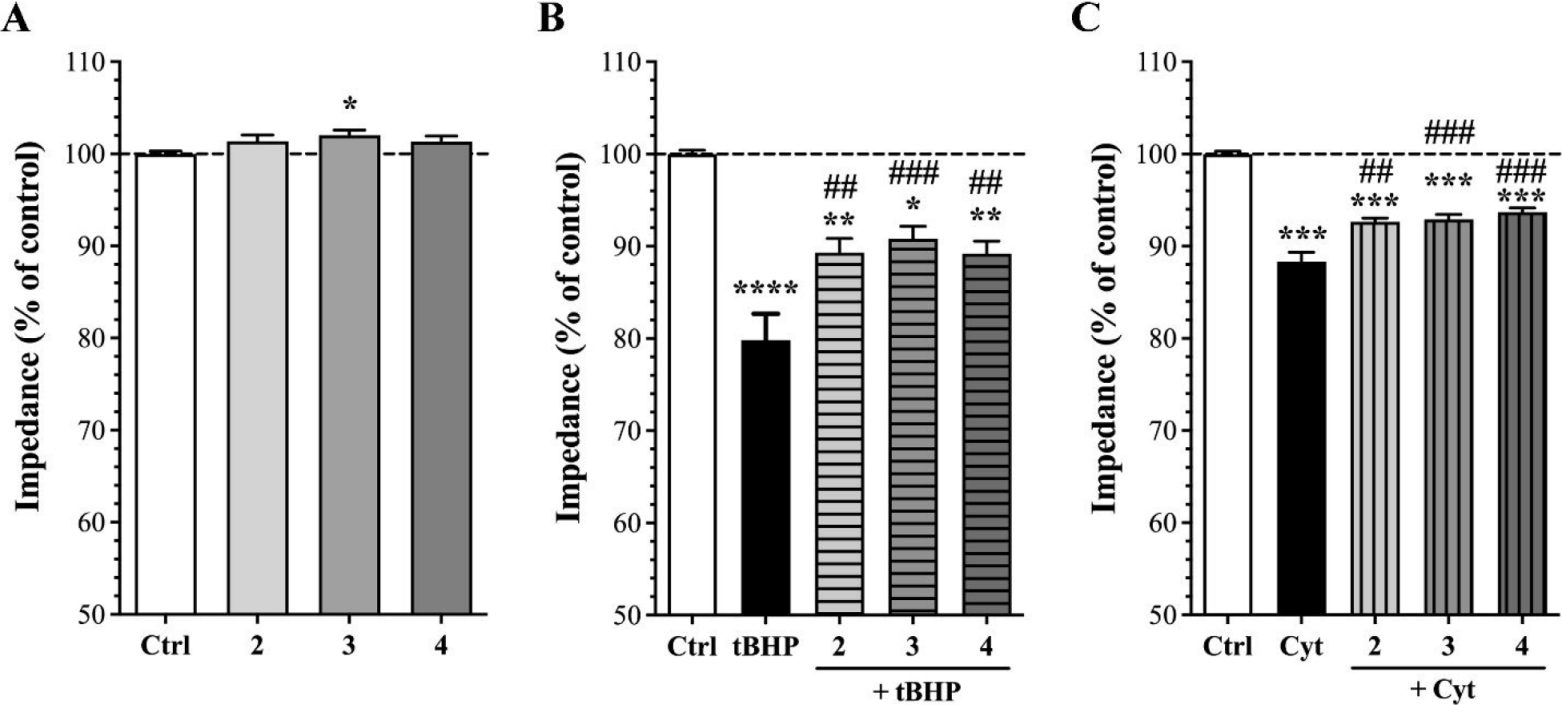
Impedance-based cell viability/barrier integrity assays to detect the protective effect of compounds **2**–**4** (1 μM, 4 h treatment) on human brain microvascular endothelial cells (hCMEC/D3) in the absence or presence of oxidative stress or cytokines. (A) Effect of compounds **2**–**4** on cell viability. (B) Effect of compounds **2**–**4** on cell impedance after cotreatment with oxidative compound *tert*-butyl hydroperoxide (tBHP, 350 μM). (C) Effect of compounds **2**–**4** on cell impedance after cotreatment with cytokines (Cyt; TNF-α and IL-1β, 10 ng/mL each). Values are presented as the mean ± standard error of the mean (SEM); tests were performed in a minimum of two independent experiments (*n* = 2–3), with 3–9 technical replicates, total *n* = 7–25. Data were analyzed by one-way analysis of variance (ANOVA) followed by Bonferroni’s multiple comparisons test. *p < 0.05, **p < 0.01, ***p < 0.001, ****p < 0.0001, compared to the control group; ^##^*p* < 0.01, ^###^*p* < 0.001, compared to the tBHP or cytokine groups.

A tendency to increase cell impedance at a 1 μM concentration by compounds **2**–**4** was observed (Figure 3A). The presence of tBHP induced a 20% significant decrease in cell viability compared to the control group (Figure 3B). Relative to this, a significant protection was observed when tBHP treatment was coadministered with compound **2**, **3**, or **4** (Figure 3B). This suggests that these compounds have a protective effect against ROS-induced cellular damage. The proinflammatory cytokines, tumor necrosis factor-α (TNF-α), and interleukin-1β (IL-1β) are involved in the increased permeability of brain endothelial cells,^17,18^ and, most recently, the presence of these cytokines has been reported around the amyloid-β plaques in the post-mortem brains of patients with Alzheimer’s disease.^19^ Therefore, to understand the protective effect of the minor ecdysteroids, compounds **2**–**4** were also tested with proinflammatory cytokines. The hCMEC/D3 cells were treated with TNF-α and IL-1β alone or in combination with the ecdysteroids. Cytokine treatment alone caused a 12% decrease in cell impedance after 4 h. This damaging effect was ameliorated with the coadministration of 1 μM of compound **2**, **3** or **4** with the cytokines (Figure 3C), indicating a protective effect against inflammation-induced barrier damage. Using similar techniques and a similar experimental paradigm, we have previously demonstrated the protective effect of the neuropeptide α-melanocyte stimulating hormone^18^ (Figure S27, Supporting Information) and grape phenolic compounds.^20^

Impedance-based monitoring of brain endothelial cell function is of high relevance not only concerning the number of viable cells but also in providing information regarding the layer’s integrity and therefore the extent of barrier damage.^18^ Accordingly, the present results suggest that the isolated minor ecdysteroids may offer potential health benefits in various central nervous system pathologies, in which the onset and/or progression of the disease is closely connected with damage to the BBB. The relevance of this finding in terms of ecdysteroid-containing food supplement consumption remains to be evaluated.

## Experimental Section

### General Experimental Procedures

Optical rotations were measured with a JASCO P-2000 polarimeter (JASCO International Co. Ltd., Hachioji, Tokyo, Japan). NMR spectra were recorded in DMSO-*d*_6_ on a Bruker Avance III 500 NMR equipped with a cryo-probehead and on a Bruker Avance III 600 spectrometer equipped with a Prodigy-probehead. Chemical shifts (δ) are given on the δ-scale and referenced to the solvent used (DMSO-*d*_6_: δ^1^H = 2.50 and δ^13^C = 39.5 ppm). The pulse programs were obtained from the Bruker software library (TopSpin 3.5). Full ^1^H and ^13^C NMR signal assignments were performed by means of comprehensive one- and two-dimensional NMR methods using widely accepted methodologies.^21,22^^1^H NMR assignments were accomplished using general knowledge of the chemical shift dispersion with the aid of the ^1^H–^1^H coupling pattern. HRMS were acquired on a Thermo Scientific Q-Exactive Plus Orbitrap mass spectrometer (Thermo Fisher Scientific Inc., Budapest, Hungary) equipped with an electrospray ionization ion source in the positive-ionization mode (HRESIMS). Flash chromatography was performed on a CombiFlash Rf+ Lumen instrument equipped with an integrated evaporative light-scattering detector (Teledyne Isco Inc., Lincoln, NE). RediSep stationary phases and flash columns were obtained from Teledyne Isco Inc. Preparative HPLC and preparative supercritical fluid chromatography (SFC) were performed on a JASCO SFC system (PU-4386 and PU-4086 pumps, MX-4300 dynamic mixer, CO-4060 thermostat, MD-4015 PDA detector, and BP-4340-H backpressure regulator; JASCO International Co. Ltd., Hachioji, Tokyo, Japan) used in HPLC or SFC mode. Centrifugal partition chromatography (CPC) was performed on an Armen Spot CPC (Armen Instrument, Saint-Avé, France) with a 250 mL multilayer coil separation column and a manual 10 mL sample loop injection valve. All reagents were purchased from Sigma-Aldrich Ltd., Hungary, unless indicated otherwise.

### Plant Material

The starting material for the isolation was a commercial extract of *Cyanotis arachnoidea* roots (20 kg) purchased from Xi’an Olin Biological Technology Co., Ltd. (Xi’an, People’s Republic of China). A representative sample of the extract was deposited at the Institute of Pharmacognosy, University of Szeged, and it is available from the authors upon request.

### Extraction and Isolation

A 5460 g sample of the starting material was extracted with methanol and subjected to an extensive multistep chromatographic isolation procedure to obtain compounds **2**–**4**; the procedure is described in detail in the Supporting Information.

#### Compound **2**

White solid, [α]^25^_D_ +68.8 (*c* 0.1, MeOH); ^13^C and ^1^H NMR data, see Table 1 and Figures S2–S7, Supporting Information; HRESIMS *m*/z 491.26435 [M + H]^+^ (calcd for C_27_H_39_O_8_^+^ 491.26394), 513.24444 [M + Na]^+^ (calcd for C_27_H_38_O_8_Na^+^ 513.24589).

#### Compound **3**

White solid, [α]^25^_D_ +54.4 (*c* 0.1, MeOH); ^13^C and ^1^H NMR data, see Table 1 and Figures S10–S16, Supporting Information; HRESIMS *m*/*z* 475.26394 [M + H]^+^ (calcd for C_27_H_39_O_7_^+^ 475.26903), 497.25116 [M + Na]^+^ (calcd for C_27_H_38_O_7_Na^+^ 497.25097).

#### Compound **4**

White solid, [α]^25^_D_ −42.9 (*c* 0.1, MeOH); ^13^C and ^1^H NMR data, see Table 1 and Figures S17–S23, Supporting Information; HRESIMS *m*/*z* 511.28879 [M + H]^+^ (calcd for C_27_H_43_O_9_^+^ 511.29016), 533.27104 [M + Na]^+^ (calcd for C_27_H_42_O_9_Na^+^ 533.27210).

### Time-Dependent Study of the Decomposition of Calonysterone

An aliquot of 10 mg of calonysterone was dissolved in 10 mL of 50% aqueous methanol and left in a sealed vial at room temperature (ca. 20–25 °C), unprotected from daylight. Analytical HPLC-PDA measurements were repeated on a weekly or biweekly basis for a total of 20 weeks, and 10 μL sample volumes were injected using a Kinetex Biphenyl column (4.6 × 250 mm, 5 μm) and an isocratic elution with 50% aqueous methanol at 1 mL/min flow rate. At each time point, the amount of compound **4** in the solution was determined based on a 9-point calibration, and the calibration line was forced to intercept at the origin (Figure S25, Supporting Information).

### Blood–Brain Barrier Cell Culture Model

The human hCMEC/D3 brain microvascular endothelial cell line^23^ was purchased from Merck Millipore (Germany). The cultures under passage number 35 were maintained in Petri dishes coated with rat tail collagen and grown in a cell culture incubator at 37 °C with 5% CO_2_. The basal medium used was MCDB 131 (Pan Biotech, Germany) supplemented with 5% fetal bovine serum (FBS), GlutaMAX (100×, Life Technologies, USA), lipid supplement (100×, Life Technologies, USA), 10 μg/mL ascorbic acid, 550 nM hydrocortisone, 37.5 μg/mL heparin, 1 ng/mL basic fibroblast growth factor (bFGF, Roche, USA), 5 μg/mL insulin, 5 μg/mL transferrin, 5 ng/mL selenium (ITS) supplement (100×, PanBiotech), 10 mM HEPES, and 50 μg/mL gentamycin. The medium was changed every 2 or 3 days. When the cultures almost reached confluence (∼90%), they were passaged to rat tail collagen-coated 96-well plates (E-plate, Agilent, USA) for the viability assays. Before each experiment, the medium was supplemented for 24 h with 10 mM LiCl to improve the BBB properties.^24^

### Cell Function Assay by Impedance Measurement

Impedance measurement correlates linearly with cell number, adherence, growth, and viability.^25^ The kinetics of the viability of brain endothelial cells after treatment was monitored by real-time impedance measurement (RTCA-SP, Agilent). The hCMEC/D3 cells were seeded at 5 × 10^3^ cells/well into the 96-well E-plate with golden electrodes at the bottom of the wells and were maintained in the CO_2_ incubator at 37 °C for 5–6 days. The medium was changed every second day. Cells at the stable plateau phase of growth were treated with compound **2**, **3** or **4** at concentrations of 0.01, 0.03, 1, 3, or 10 μM. Triton X-100 detergent was used to determine 100% toxicity. The effects of the treatment were observed for 48 h.

### Preparation of Stock and Working Solutions for the Cellular Assays

All compounds were produced in powder form. The stock solutions were prepared by diluting the compounds in dimethyl sulfoxide (DMSO) at a final concentration of 10 mM. The compounds were stored at −20 °C. Aliquots were always freshly thawed, and a dilution was made in the cell culture medium to have a 100 μM solution. From these 100 μM solutions, serial dilutions were made to prepare the working solutions at the following concentrations: 10, 3, 1, 0.3, 0.1, 0.03, and 0.01 μM. The 1 μM concentration was determined to be the most effective for all the compounds and was used for further tests.

### Cytokine Treatment

A combination of human TNF-α (10 ng/mL) and human IL-1β (10 ng/mL; Peprotech, USA) was added to the cell cultures to promote inflammatory reactions.^26^ To determine if the compounds had an anti-inflammatory characteristic, cytokine treatment was combined with the selected concentration of compound **2**, **3**, or **4**. The control groups received cell culture medium while another group was treated with cytokines only.

### Induction of Oxidative Stress

tBHP is an oxidative compound that may induce cell death via apoptosis or necrosis. tBHP generates *tert*-butoxyl radicals via iron-dependent reactions, resulting in lipid peroxidation and depletion of intracellular glutathione followed by the modification of protein thiols resulting in the loss of cell viability.^16,27,28^ Various concentrations were tested to determine a ca. 50% cell viability loss. In the preliminary experiments, concentrations from 1 to 1000 μM were tested, and 350 μM tBHP was found to be effective and optimal to reduce cell viability to 50%. Therefore, this concentration was combined with the selected concentrations of the compounds used to test for potential protective effects.

### Statistics

Data are presented as means ± standard error of the mean (SEM). Statistical significance between treatment groups was determined using one-way analysis of variance (ANOVA) followed by Dunnett’s or Bonferroni multiple comparison post-tests (GraphPad Prism 9.0; GraphPad Software, USA). A minimum of four parallel samples were tested. Changes were considered statistically significant at *p* < 0.05.

## Supporting Information Available

Detailed experimental procedure for the chromatographic isolation of compounds **2**–**4**, high-resolution mass spectra and characteristic one- and two-dimensional NMR spectra for compounds **2**–**4**, HPLC-PDA fingerprints for the decomposition process of calonysterone to compound **4**, calibration for compound **4**, and the effect of compounds **2**–**4** on human brain microvascular endothelial cells’ impedance (PDF)
