# Correction to “Highly Oxidized Ecdysteroids from a Commercial *Cyanotis arachnoidea* Root Extract as Potent Blood–Brain Barrier Protective Agents”

**DOI:** 10.1021/acs.jnatprod.3c00927

**Published:** 2023-11-23

**Authors:** Gábor Tóth, Ana R. Santa-Maria, Ibolya Herke, Tamás Gáti, Daniel Galvis-Montes, Fruzsina R. Walter, Mária A. Deli, Attila Hunyadi

Page 1075: The authors are reporting a correction to the ^1^H NMR chemical shifts of compound **3**. The previously published table contains a systematic copy-paste type error that originates from the chemical shifts of compound **4**. The authors sincerely apologize for any inconvenience caused by this error. Corrected Table 1 is provided below.

**Table 1:** ^1^H and ^13^C NMR Chemical Shifts of Compounds **1**–**4** in DMSO-*d*_6_

	**1**^*a*^		**2**^*a*^		**3**^*a*^		**4**^*b*^	
no.	^1^H	^13^C	^1^H	^13^C	^1^H	^13^C	^1^H	^13^C
1β	2.17	40.3	2.17	41.1	1.25	41.5	1.64	34.8
α	1.14		1.13		2.27		2.17	
2α	3.80	68.0	3.81	68.0	3.83	68.1	3.91	68.6
3α	3.31	71.7	3.33	71.9	3.33	72.0	3.39	69.3
4β	2.35	26.8	2.36	26.8	2.37	26.8	2.30	26.8
α	2.89		2.93		2.91		2.80	
5		133.2		133.7		132.8		136.9
6		142.6		142.6		142.6		142.8
7		180.2		179.1		179.4		185.2
8		131.5		124.8		122.7		129.9
9		161.7		161.3		163.9		73.7
10		40.5		40.76		40.8		42.2
11β	2.32	22.4	∼2.34	21.4	6.72	125.7	2.12	27.0
α	2.22		∼2.34				1.54	
12β	1.72	35.0	1.56	34.0	2.16	35.8	1.96	31.5
α	1.37		1.07		1.44		1.62	
13		40.4		35.5		45.9		46.7
14β	2.43	46.4	4.05	50.7		141.0		169.5
15β	2.08	30.2		212.5	∼2.55	23.7	4.58	68.9
α	0.92				∼2.55			
16β	1.70	25.2		80.1	2.62	31.7	2.21	31.7
α	1.50		4.10		1.91		1.57	
17α	1.68	50.8	2.34	57.6	2.06	56.1	1.93	51.5
18	1.10	23.8	1.22	17.1	0.92	17.3	1.02	18.8
19	1.36	26.5	1.35	27.0	1.40	27.1	1.20	27.3
20		75.6		78.8		79.8		75.2
21	1.13	19.9	1.31	23.6	1.31	24.6	1.12	20.9
22	3.20	76.4	3.33	89.9		217.3	3.23	76.2
23	1.42	26.0	1.51	22.2	2.71	32.1	1.46	26.1
	1.13		1.43		2.71		1.12	
24	1.64	41.3	1.57	40.8	∼1.51	37.0	1.63	41.3
	1.13		1.31		∼1.51		1.26	
25		68.7		68.7		68.1		68.8
26	1.03	29.0	1.07	29.1	1.07	29.3	1.04	29.2
27	1.05	29.9	1.08	29.7	1.07	29.34	1.06	29.9
HO-2	4.57		4.59		4.62		4.43	
HO-3	4.87		4.93		4.92		4.77	
HO-6	8.02		8.15		8.07		7.89	
HO-9							4.94	
HO-15							5.39	
HO-20	3.61		4.73		5.02		3.76	
HO-22	4.33						4.45	
HO-25	4.07		4.13		4.19		4.15	

