# Supplementary material for: Highly Oxidized Ecdysteroids from a Commercial Cyanotis arachnoidea Root Extract as Potent Blood–Brain Barrier Protective Agents
Source: J Nat Prod. 2023 Feb 24;86(4):1074–80. doi: 10.1021/acs.jnatprod.2c00948 (PMC10152481; doi:10.1021/acs.jnatprod.2c00948)
Supplement: Supplementary file 1 — np2c00948_si_001.pdf [file np2c00948_si_001.pdf]

Supporting information for

Highly Oxidized Ecdysteroid Derivatives from a Commercial *Cyanotis arachnoidea* Root Extract as Potent Blood-Brain Barrier Protective Agents

Gábor Tóth,[†] Ana R. Santa-Maria,[‡,§] Ibolya Herke,[††] Tamás Gáti,[⊥] Daniel Galvis-Montes,[‡] Fruzsina R. Walter,[‡] Mária A. Deli,*[,‡] and Attila Hunyadi*[,††,∥]

[†] Department of Inorganic and Analytical Chemistry, NMR Group, Budapest University of Technology and Economics, H-1111 Budapest, Hungary

[‡] Institute of Biophysics, Biological Research Centre, Szeged, H-6726 Hungary

[§] Wyss Institute for Biologically Inspired Engineering at Harvard University, Boston, MA 02115, United States

[⊥] Servier Research Institute of Medicinal Chemistry (SRIMC), H-1031 Budapest, Hungary

[††] Institute of Pharmacognosy, and [∥] Interdisciplinary Centre of Natural Products, University of Szeged, H-6720 Szeged, Hungary

*: Corresponding authors, email: hunyadi.attila@szte.hu (A.H.) and deli.maria@brc.hu (M.D.)

Table of Contents

Chromatographic Isolation Procedure for Compounds **2–4** 3

Figure S1. Compound **2**, HRMS 5

Figure S2. Compound **2**, ^{1}H NMR, DMSO-d$_6$, Bruker Avance III cryo 6

Figure S3. Compound **2**, sel-Roesy (τ_{mix}: 300 ms) on Me-19, -18, and -21 6

Figure S4. Compound **2**, sel-Roesy (τ_{mix}: 300 ms) on Hα-16, Hβ-14 and Hα-22+H-3 7

Figure S5. Compound **2**, DEPTQ 7

Figure S6. Compound **2,** edHSQC section 8

Figure S7. Compound **2**, HMBC and HMBC section 8

Figure S8. Compound **3**, HRMS 9

Figure S9. Compound **3,** ^{1}H, DMSO-d$_6$ Bruker Avance III cryo 500 MHz 10

Figure S10. Compound **3**, selTOCSY on H-2, Hα-4, Hα-17 and Hα-16 10

Figure S11. Compound **3**, selROE on H_3-19, H_3-21 and H_3-18 11

Figure S12. Compound **3**, DEPTQ 11

Figure S13. Compound **3**, HSQC 12

Figure S14. Compound **3**, edHSQC CH_2 section 12

Figure S15. Compound **3**, HMBC and HMBC section 13

Figure S16. Compound **4,** HRMS 14
Figure S17. Compound **4**, ^{1}H DMSO-d_6 600 MHz 15
Figure S18. Compound **4**, selTOCSY (τ_{mix}: 80 ms) on H-15, H-2 and H-22 15
Figure S19. Compound **4**, sel-Roesy (τ_{mix}: 300 ms) on Me-18, Me-21 and Me-19 16
Figure S20. Compound **4**, DEPTQ 16
Figure S21. Compound **4**, edHSQC 17
Figure S22. Compound **4**, edHSQC section 17
Figure S23. Compound **4**, HMBC and HMBC section 18
Figure S24. Gradual decomposition of calonysterone (**Cal**) to compound **4** in 50% aqueous methanol. 18
Figure S25. Calibration for the determination of compound **4**. 19
Figure S26. Effect of compounds **2**–**4** on human brain endothelial cell viability 20
Figure S27. Impedance-based cell viability/barrier integrity assay to detect the effects of α-melanocyte stimulating hormone (α-MSH) on cytokine treated rat brain endothelial cells. . 20

Chromatographic Isolation Procedure for Compounds 2–4

A multi-step chromatographic isolation was performed. Solvent system compositions described below are always given in volumetric ratios. An aliquot of 5460 g of CA was percolated with 15.5 L of MeOH the extract and evaporated to dryness under reduced pressure to yield 1398 g of dry residue. A 700 g aliquot of this extract was adsorbed on 2100 g of silica and applied on top of a column of 1500 g of silica. A stepwise gradient was performed, eluting with CH_2Cl_2 (20 L) and CH_2Cl_2 – MeOH mixtures (97:3, 10 L; 95:5, 20 L; 93:7, 22.5 L; 85:15, 25 L). After evaporating to dryness under vacuo, the fractions eluted with 95:5, and those with 93:7 gave 210 (F1), and 415.8 g (F2) of dry residues, respectively.

Fraction F1 was adsorbed to 840 g of Celite 545 and layered on top of a 2540 g silica column. Gradient elution was performed, and a total of 80 fractions of 2.5 L each were collected, using *c*-hexane (fr. 1), c-hexane – ethyl acetate (6:2, fr. 2-3; 6:3, fr. 4-5; 6:4, fr. 6-9; and 6:5, fr.10-19), and *c*-hexane – ethyl acetate – ethanol (60:50:2, fr. 20-30; 60:50:5, fr. 31-49; 60:50:10, fr. 50-66; and 60:50:20, fr. 67-80) solvent systems. Fractions 26-51 (130.8 g) were combined, adsorbed on 327 g of RediSep Rf C_{18} (40-60 um, Teledyne Isco Inc, Lincoln, NE, USA), applied on top of 400 g of the same stationary phase, and further purified by a stepwise gradient of aqueous acetonitrile (20%, fr. 1-23; 30%, 24-31; and 40%, 32-35). The dry residue of fractions 6-10 (29.8 g) was fractionated by centrifugal partition chromatography (CPC) in multiple steps, using *n*-hexane – ethyl acetate – methanol – water (3:7:3:7) in ascending mode. Fraction collection was monitored by the instrument's dual-wavelength UV detector, and the first significant peak gave a fraction containing one chief constituent. This was purified by preparative HPLC (Kinetex Biphenyl; 21x250 mm, 5µm; 55% MeOH(aq), flow rate: 15 mL/min) to afford compound **3** (40.0 mg).

Fraction F2 (415.8 g) was adsorbed on 1000 g of silica, applied on top of a column of 2000 g of silica, and further fractionated with a stepwise gradient of *n*-hexane – ethyl acetate – ethanol, 60:40:0, 60:50:0, 60:50:2, 60:50:5, 60:50:10, 60:50:20, and 60:50:30, collecting 350 fractions of 500 mL each (F2.1-350). Fractions F2.81-94 (115.93 g) were joined, adsorbed onto 600 g of silica, applied on top of a column of 700 g of silica, and further fractionated with a stepwise gradient of *n*-hexane – ethyl acetate – isopropanol – water (100:100:2:1, fr. 1-14; 100:100:10:2, fr. 15-40; and 100:100:20:2). Fractions 52-62 (20.8 g) were joined, adsorbed on 75 g of RediSep Rf C_{18} (40-60 um, Teledyne Isco Inc, Lincoln, NE, USA), applied on top of 460 g of the same stationary phase, and further fractionated with a stepwise gradient of aqueous methanol (30%, fr. 1-28; 35%, fr. 29-61; 40%, fr. 62-111; 50%, fr. 112-127; and 100%, fr. 128-136). From the joint fractions 43-63 calonysterone was crystallized, and the mother liquid was purified by preparative HPLC (Kinetex Biphenyl; 21x250 mm, 5µm; 45% MeOH(aq), flow rate: 10 mL/min) to afford compound **4** (20 mg).

Fractions F2.115-153 (78.9 g) were joined, adsorbed on 300 g of RediSep Rf C_{18} (40-60 um, Teledyne Isco Inc, Lincoln, NE, USA), applied on top of 500 g of the same stationary phase, and further fractionated with aqueous methanol (10%, fr. 1-37; 15%, fr. 38-50; 20%, fr. 51-60; 25%, fr. 61-83; 30%, fr. 84-97; and 40%, fr. 98-127). Fractions 91-105 (10.4 g), 106-115 (7.7 g), and 116-127 (4.9 g) were separately purified by flash chromatography (dry loading on 20 g of silica, 330 g RediSep Silica Gold column, solvent A: CH_2Cl_2, solvent B: 99% methanol(aq), gradient: 4% B in A increasing to 10% in 60 min). Combining the first eluting peak of each separation gave 200 mg of a mixture containing one chief constituent that was crystallized to obtain compound **2**.

Figure S1. Compound **2**, HRMS

$C_{27}H_{38}O_8$

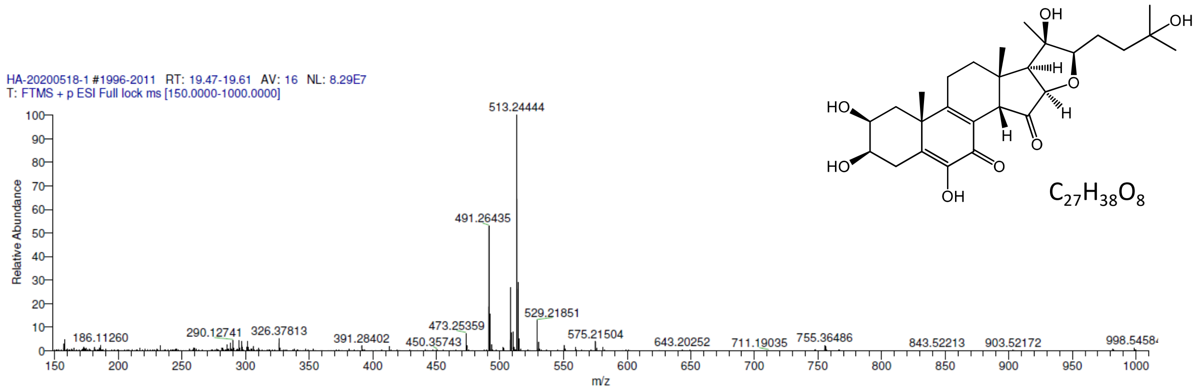

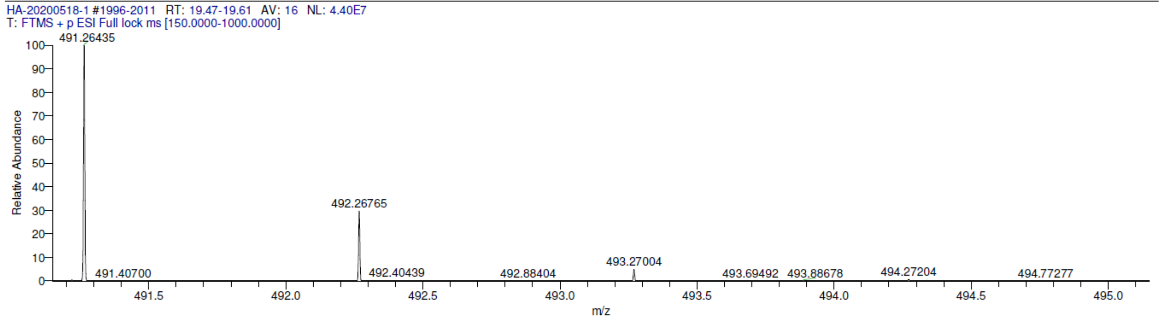

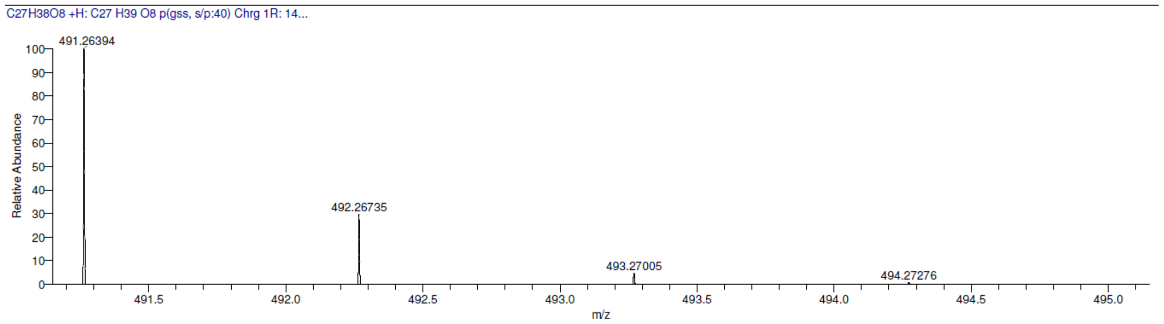

Figure S2. Compound **2**, ^{1}H NMR, DMSO-d_6, Bruker Avance III cryo

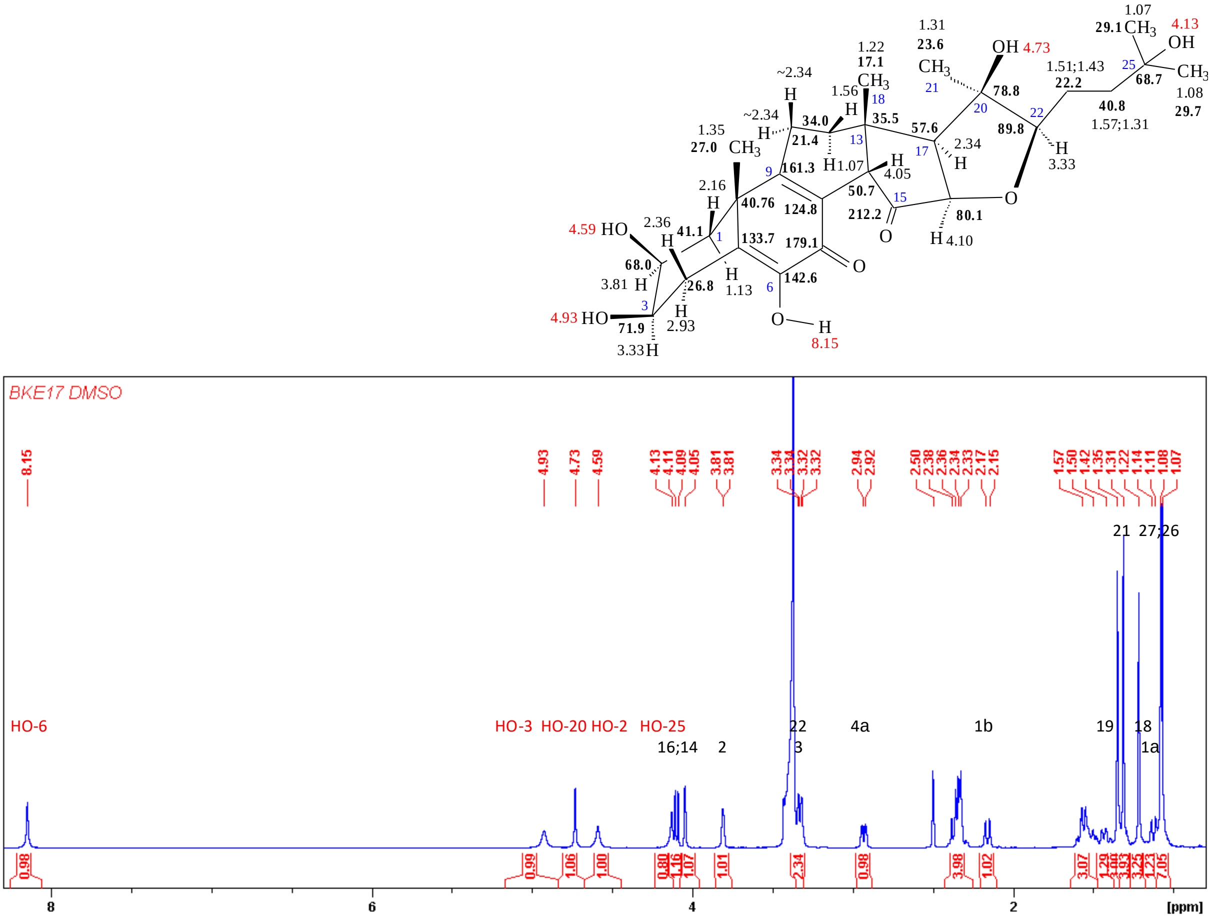

Figure S3. Compound **2**, sel-Roesy (τ_{mix}: 300 ms) on Me-19, -18, and -21

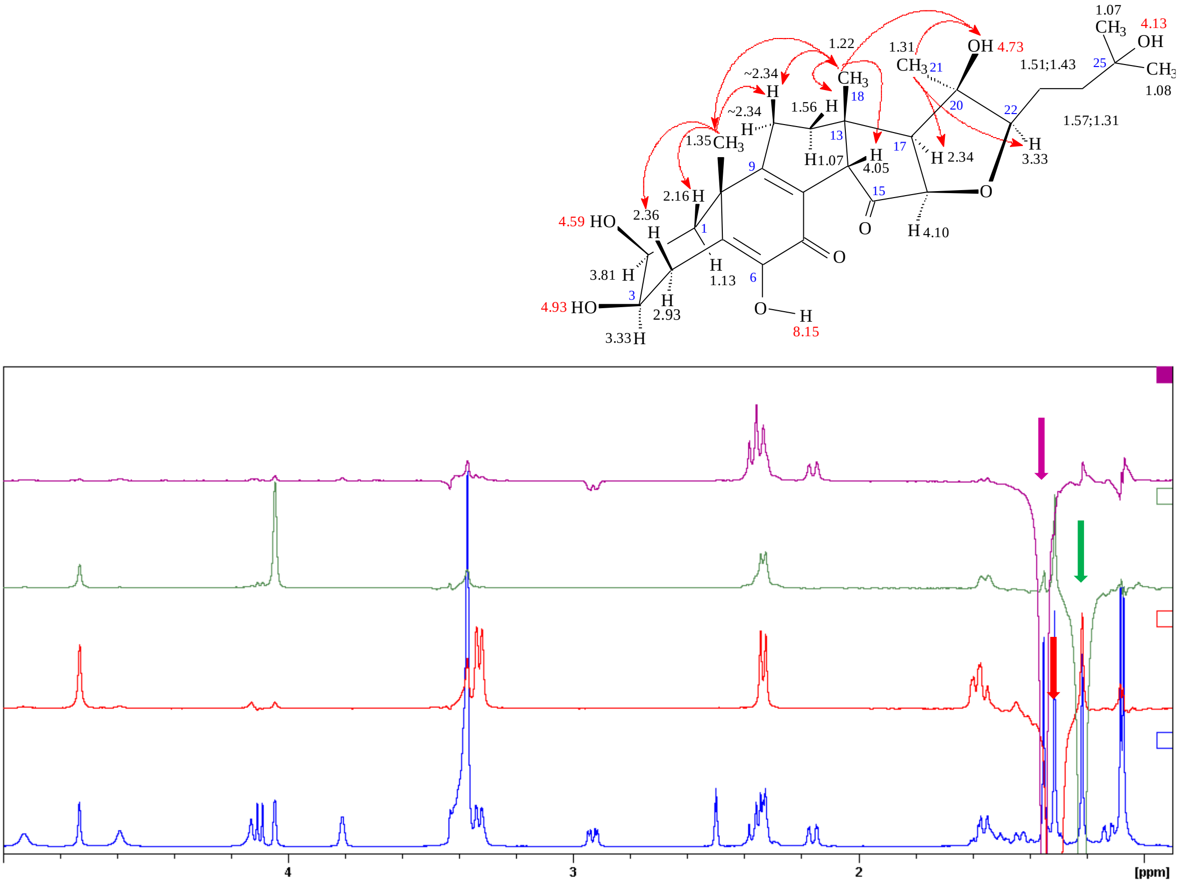

Figure S4. Compound **2**, sel-Roesy (τ_{mix}: 300 ms) on Hα-16, Hβ-14 and Hα-22+H-3

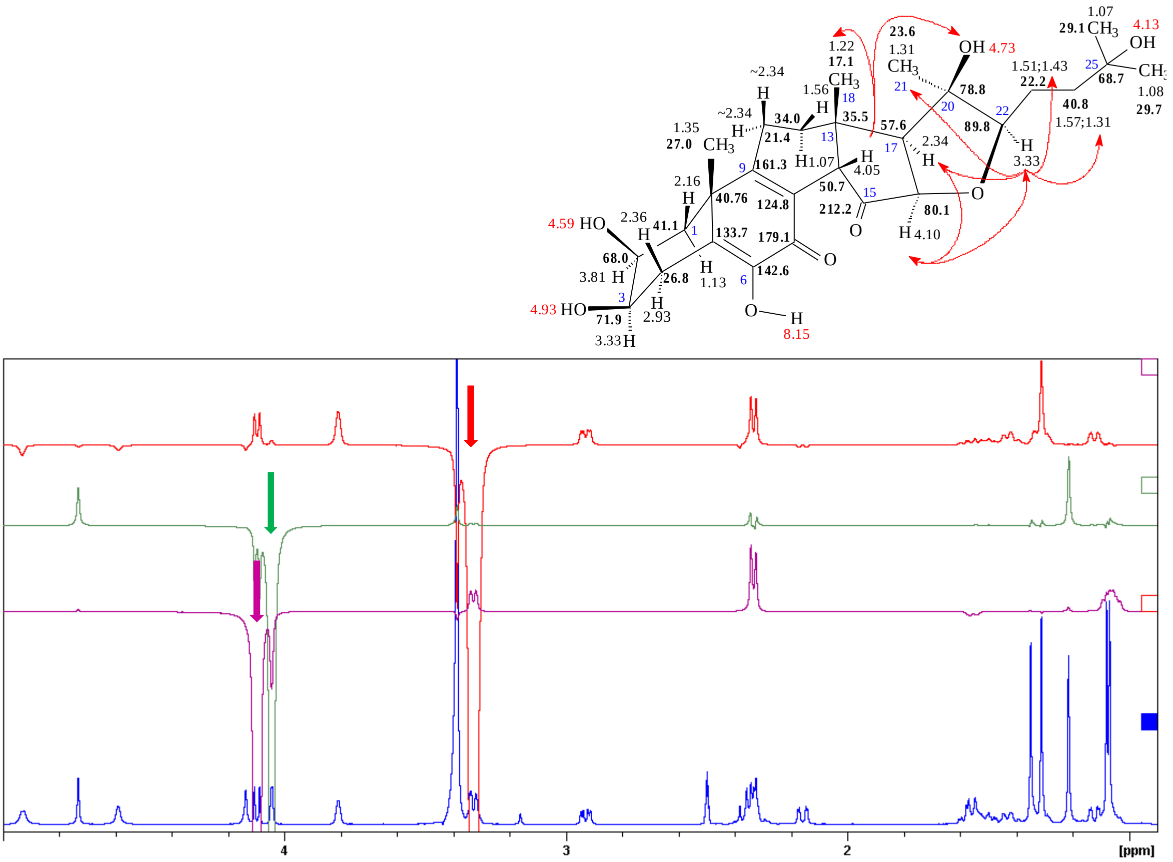

Figure S5. Compound **2**, DEPTQ

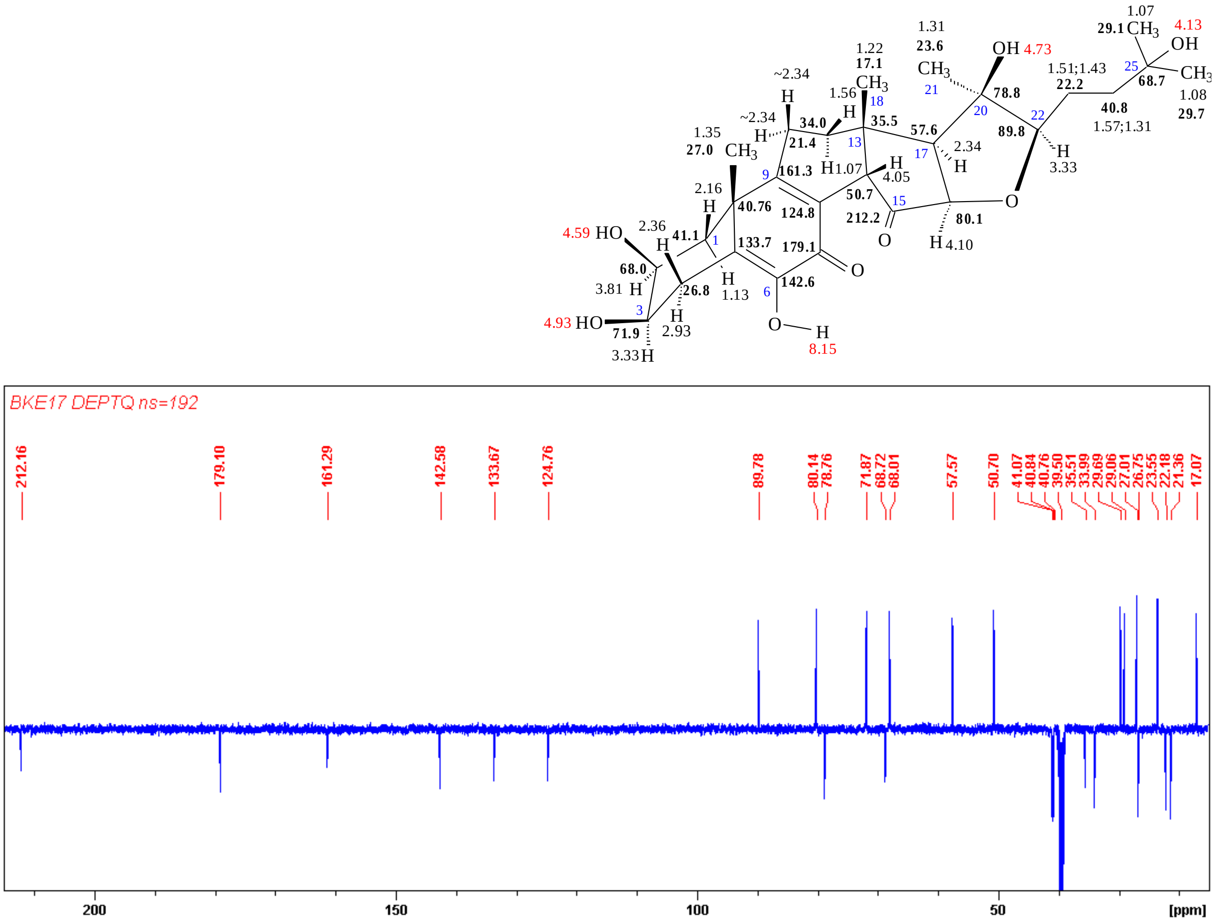

Figure S6. Compound **2**, edHSQC section

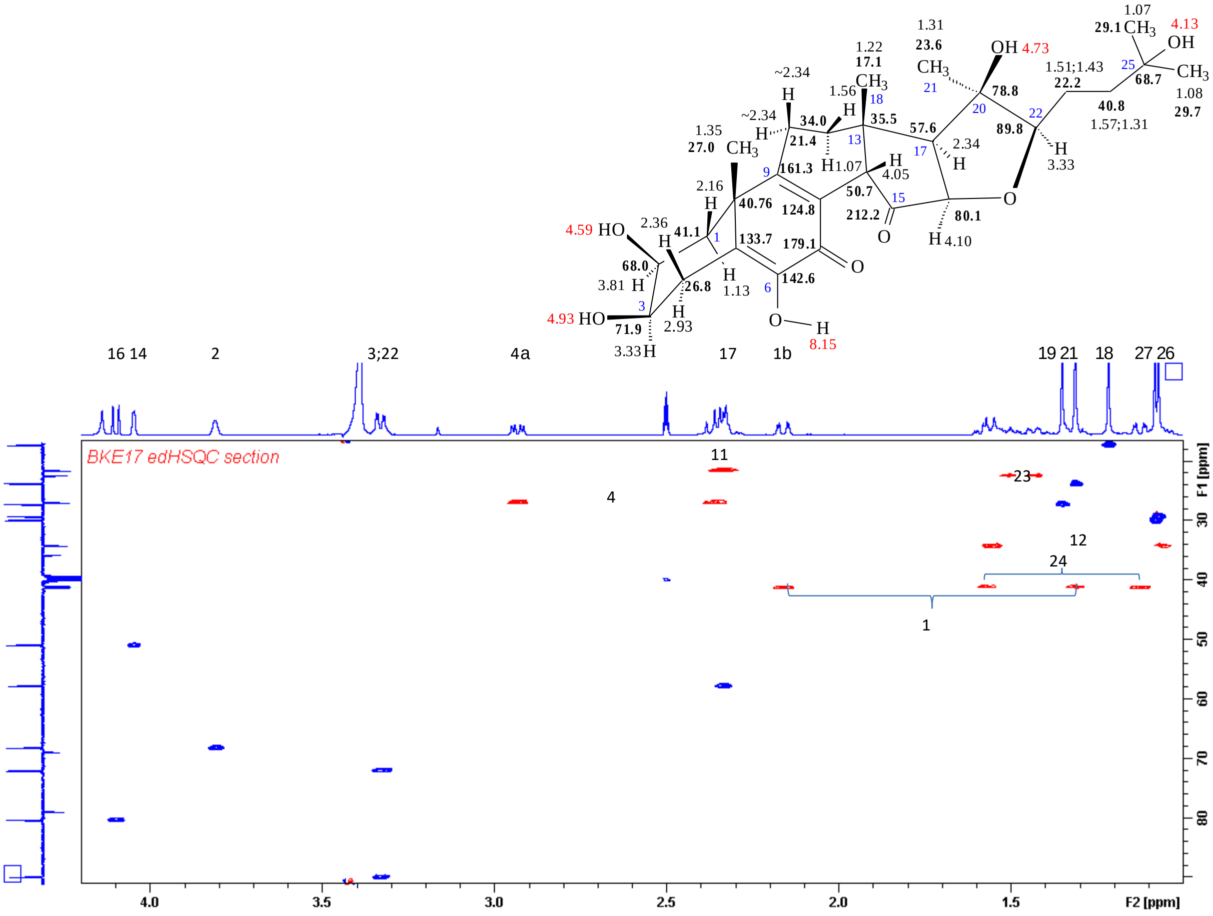

Figure S7. Compound **2**, HMBC and HMBC section

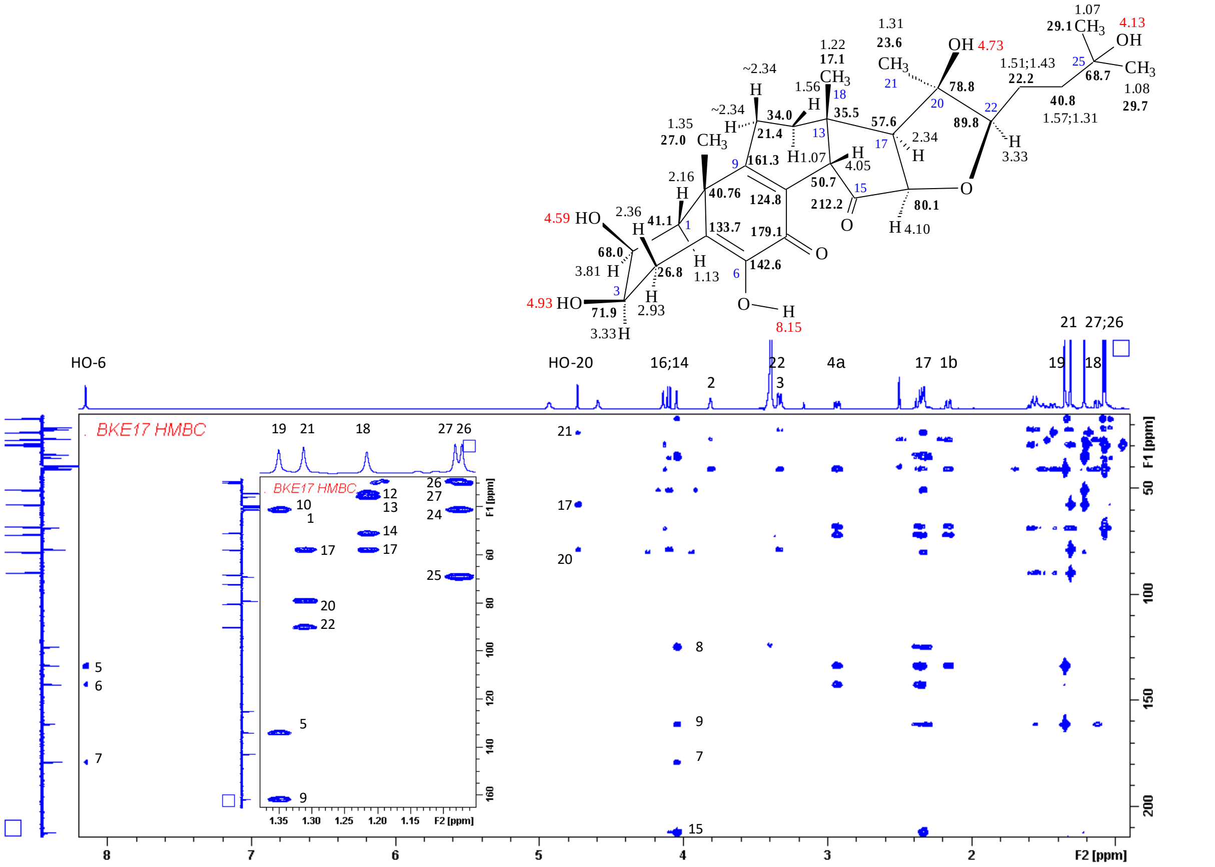

Figure S8. Compound **3**, HRMS

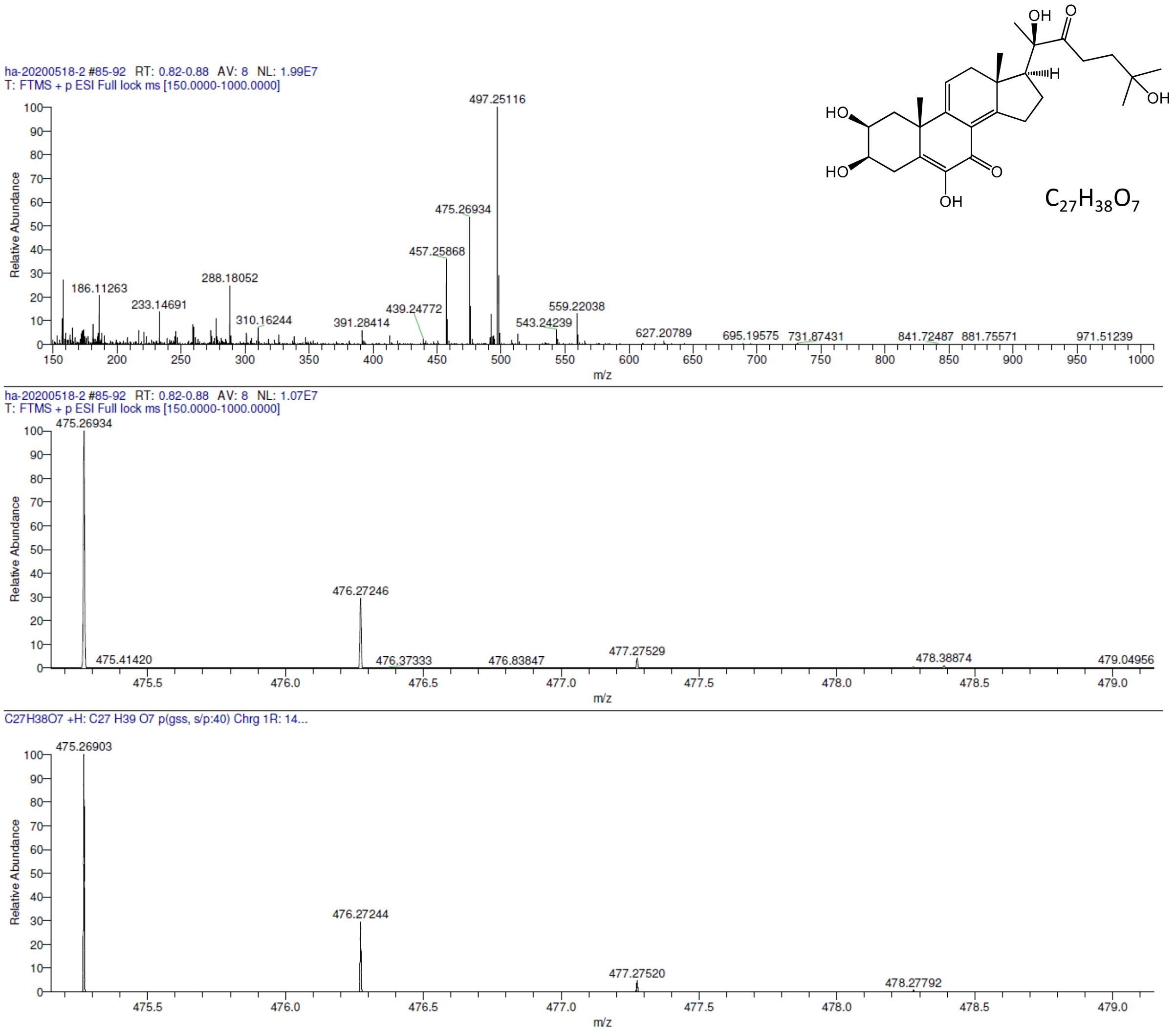

Figure S9. Compound **3**, ^{1}H, DMSO-d$_6$ Bruker Avance III cryo 500 MHz

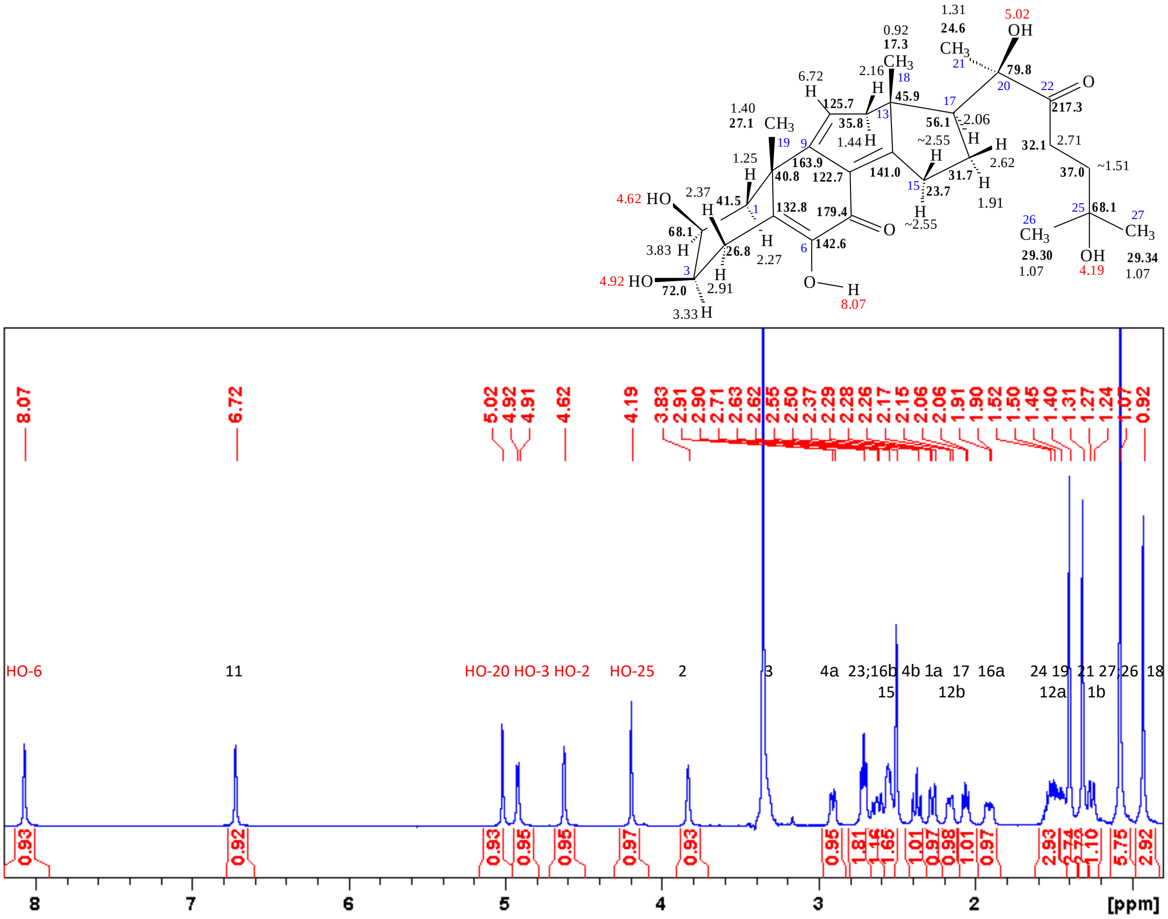

Figure S10. Compound **3**, selTOCSY on H-2, Hα-4, Hα-17 and Hα-16

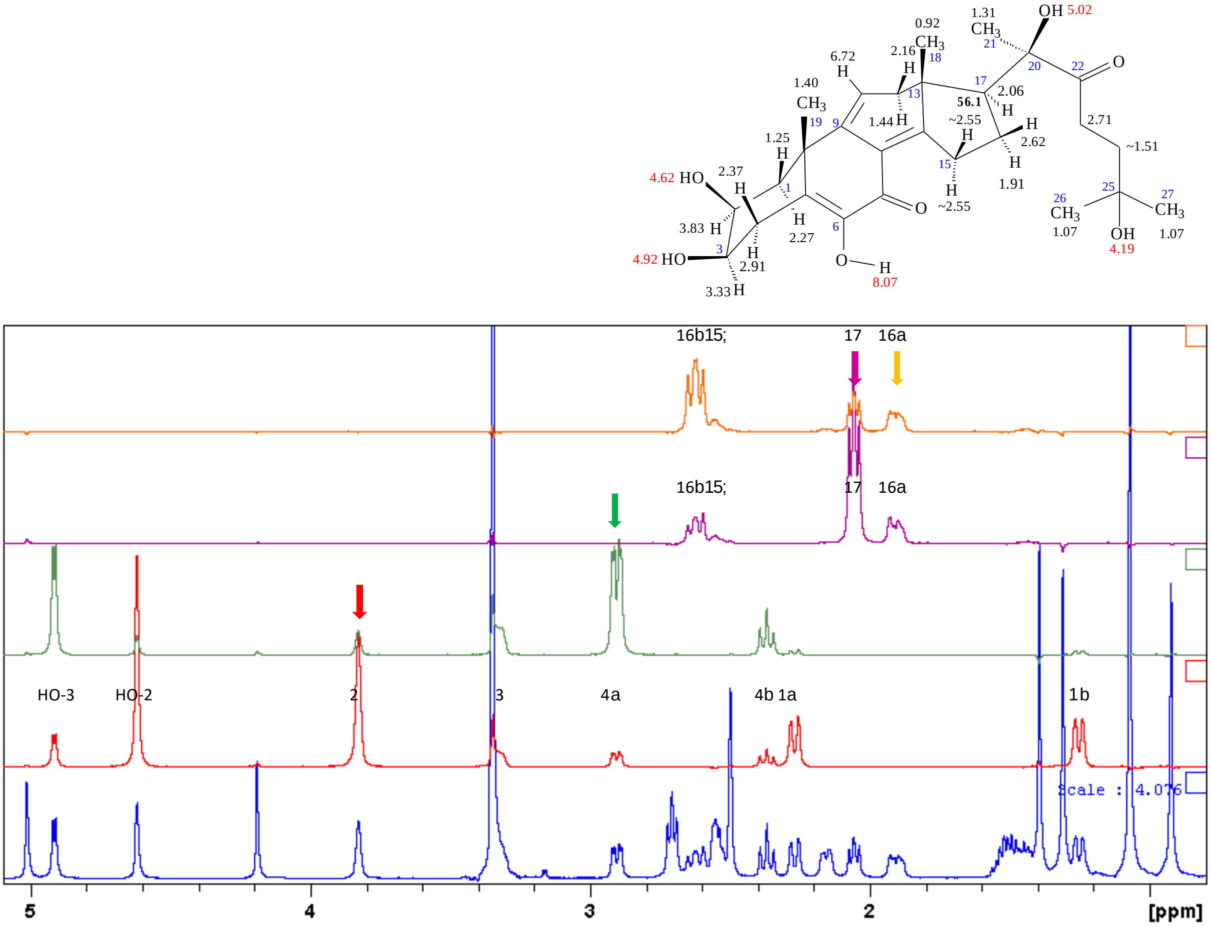

Figure S11. Compound **3**, selROE on H_3-19, H_3-21 and H_3-18

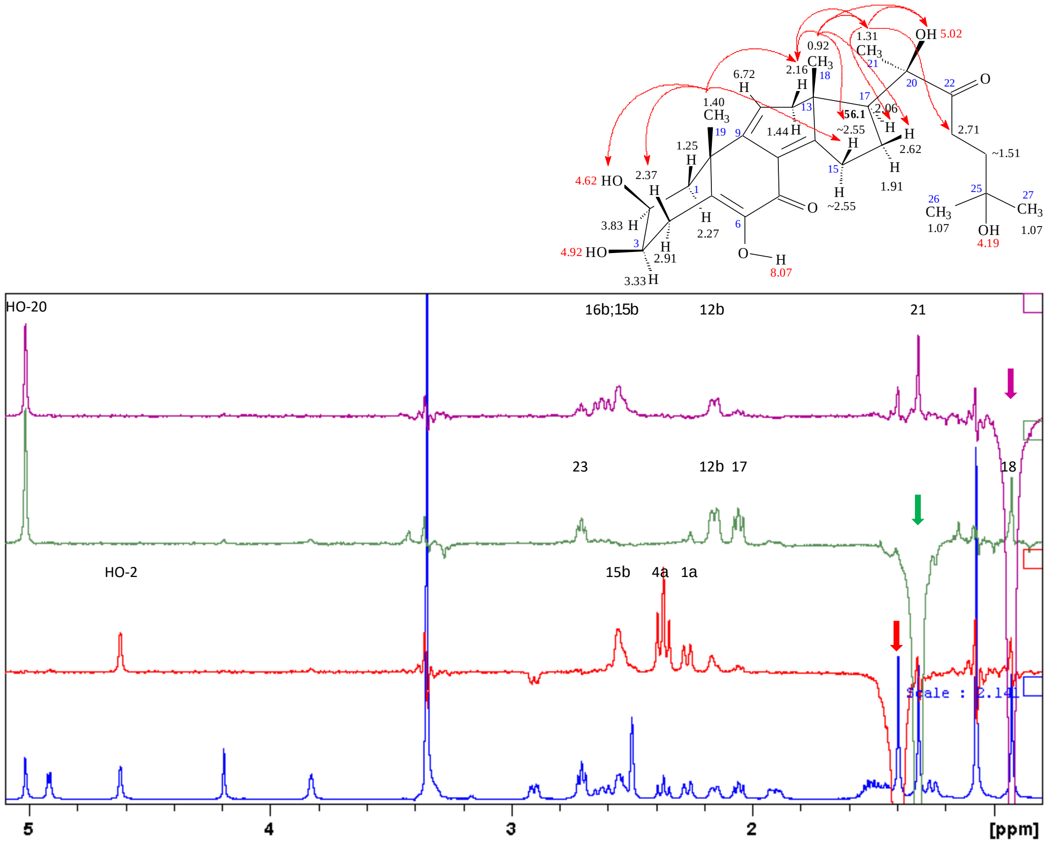

Figure S12. Compound **3**, DEPTQ

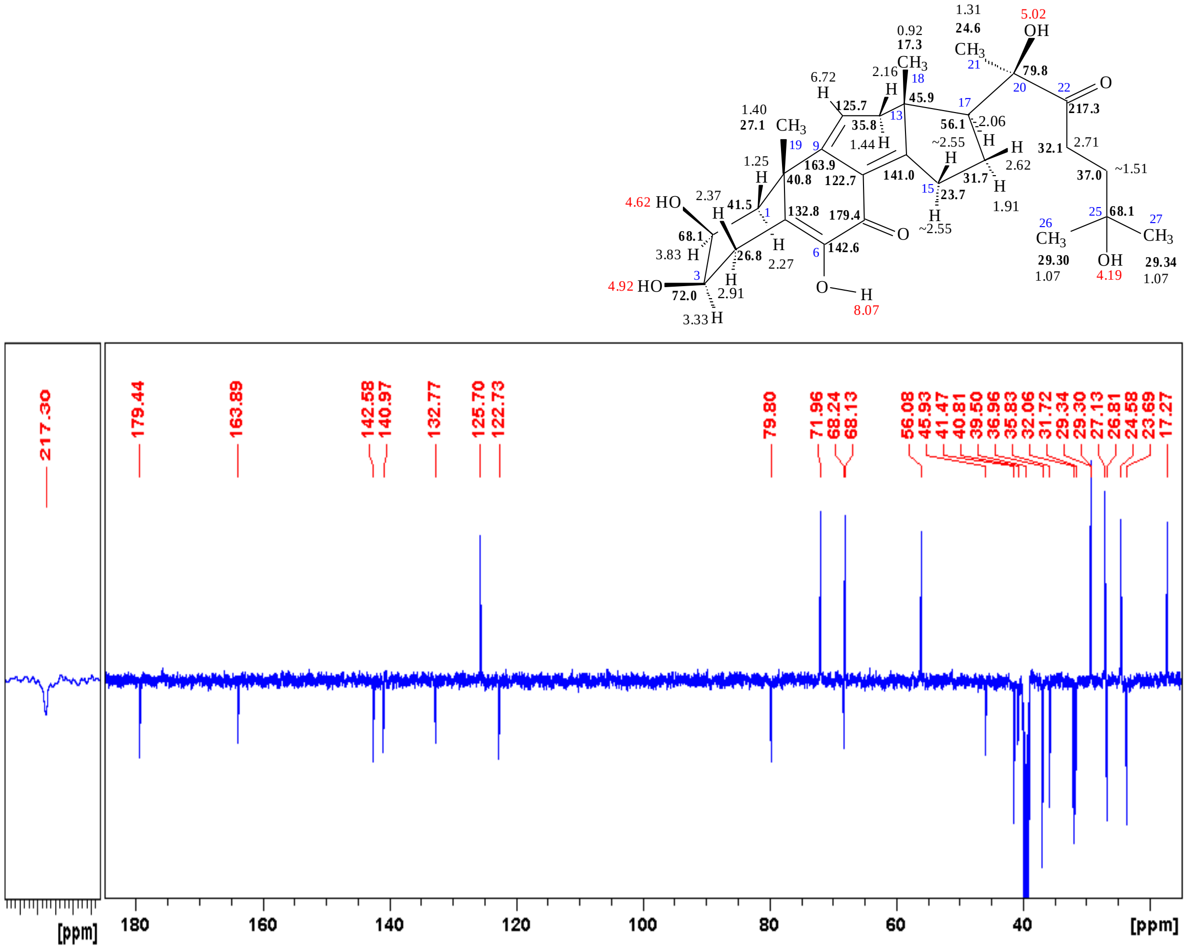

Figure S13. Compound **3**, HSQC

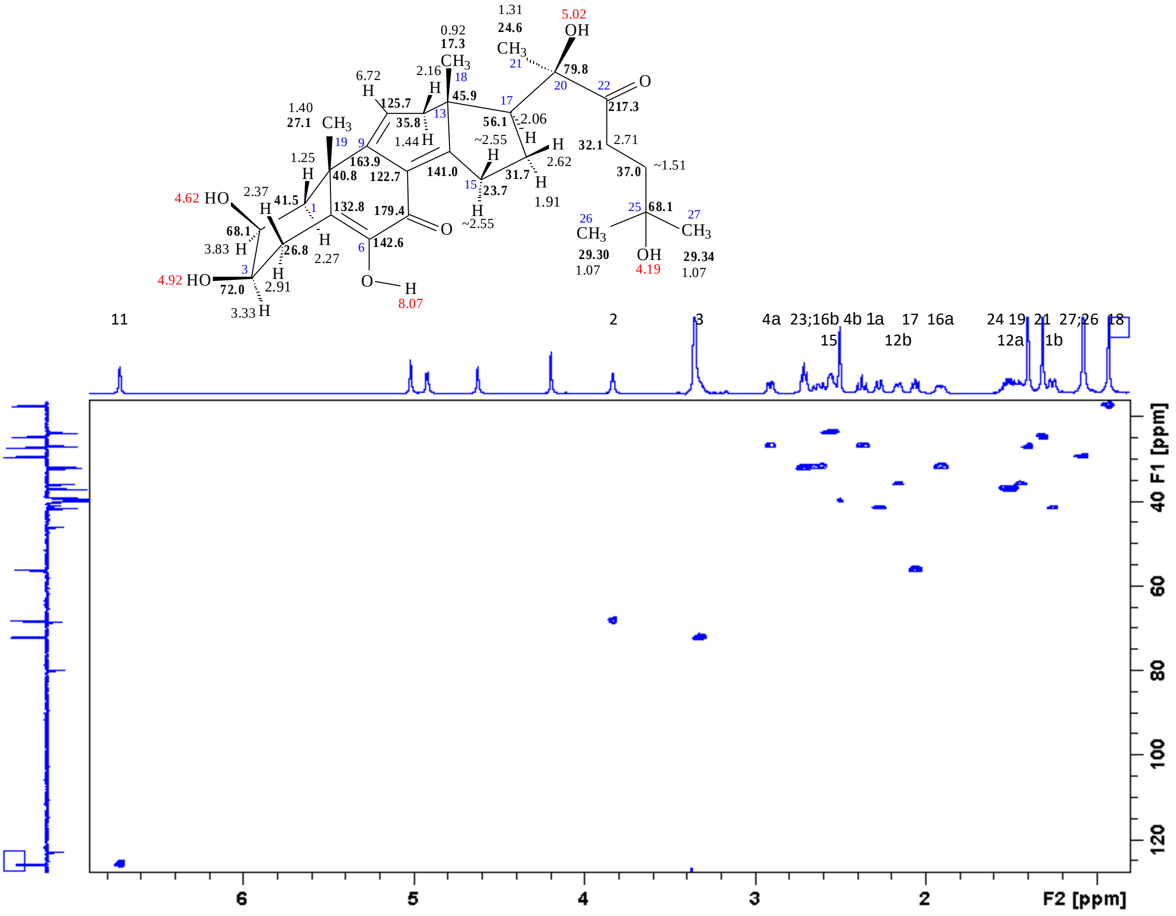

Figure S14. Compound **3**, edHSQC CH_2 section

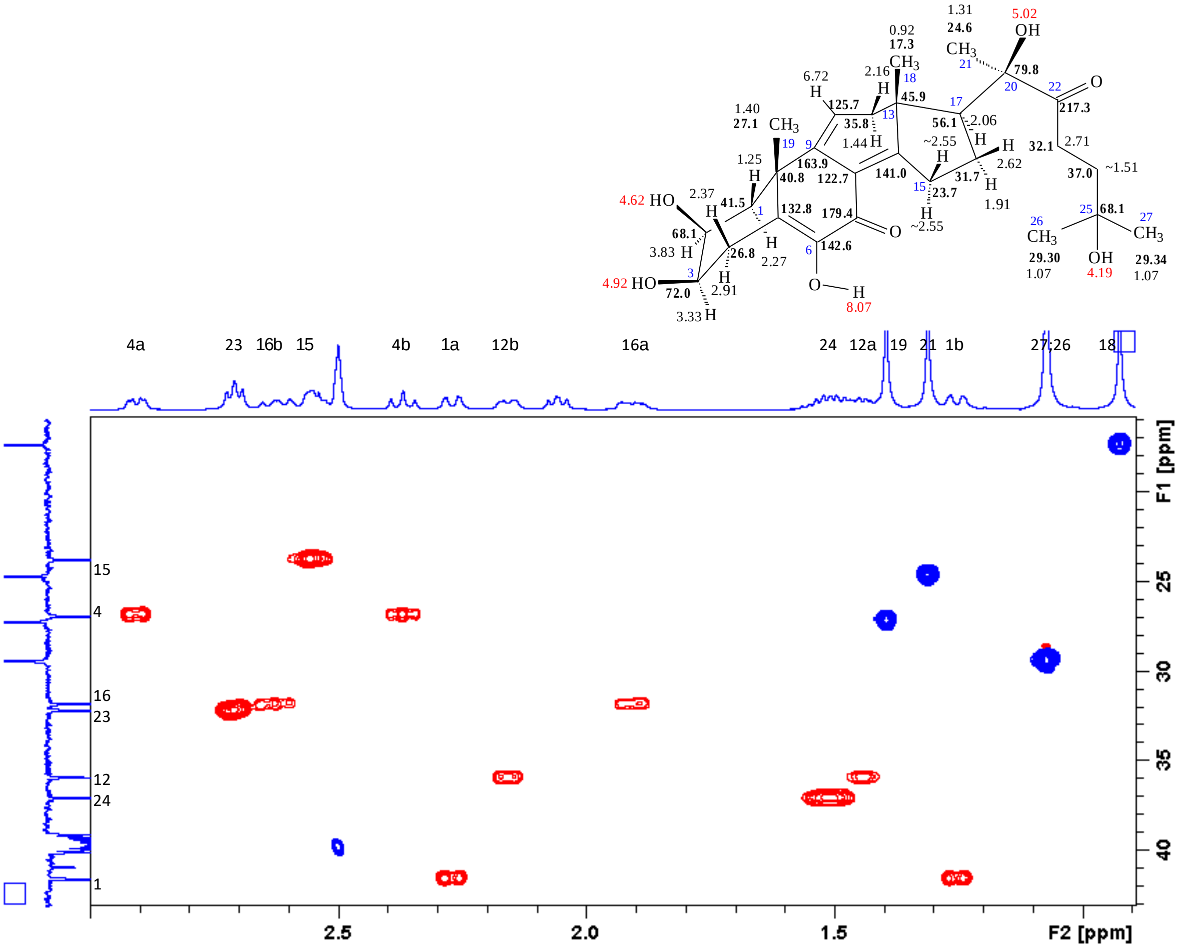

Figure S15. Compound **3**, HMBC and HMBC section

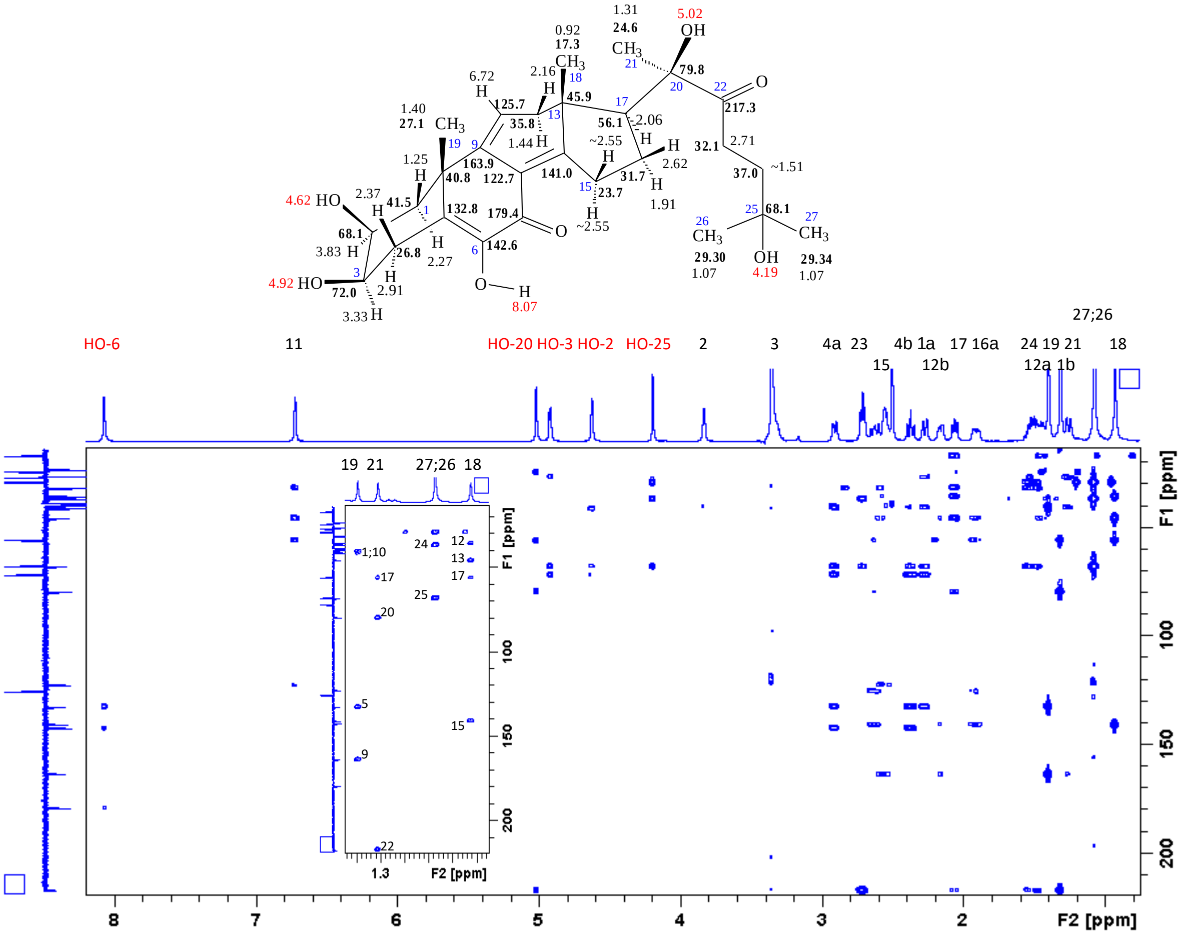

Figure S16. Compound **4**, HRMS

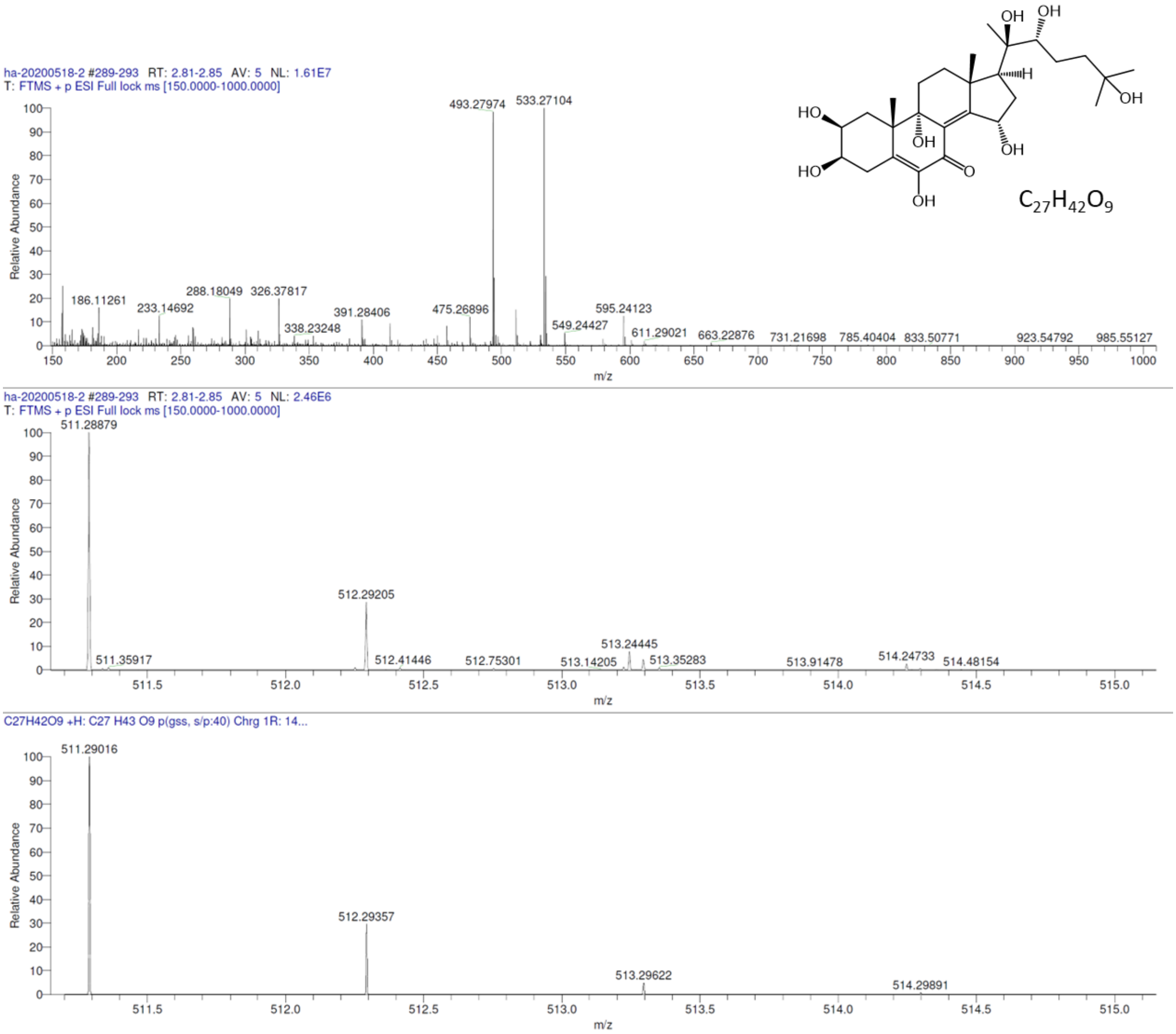

Figure S17. Compound **4**, ^{1}H DMSO-d$_6$ 600 MHz

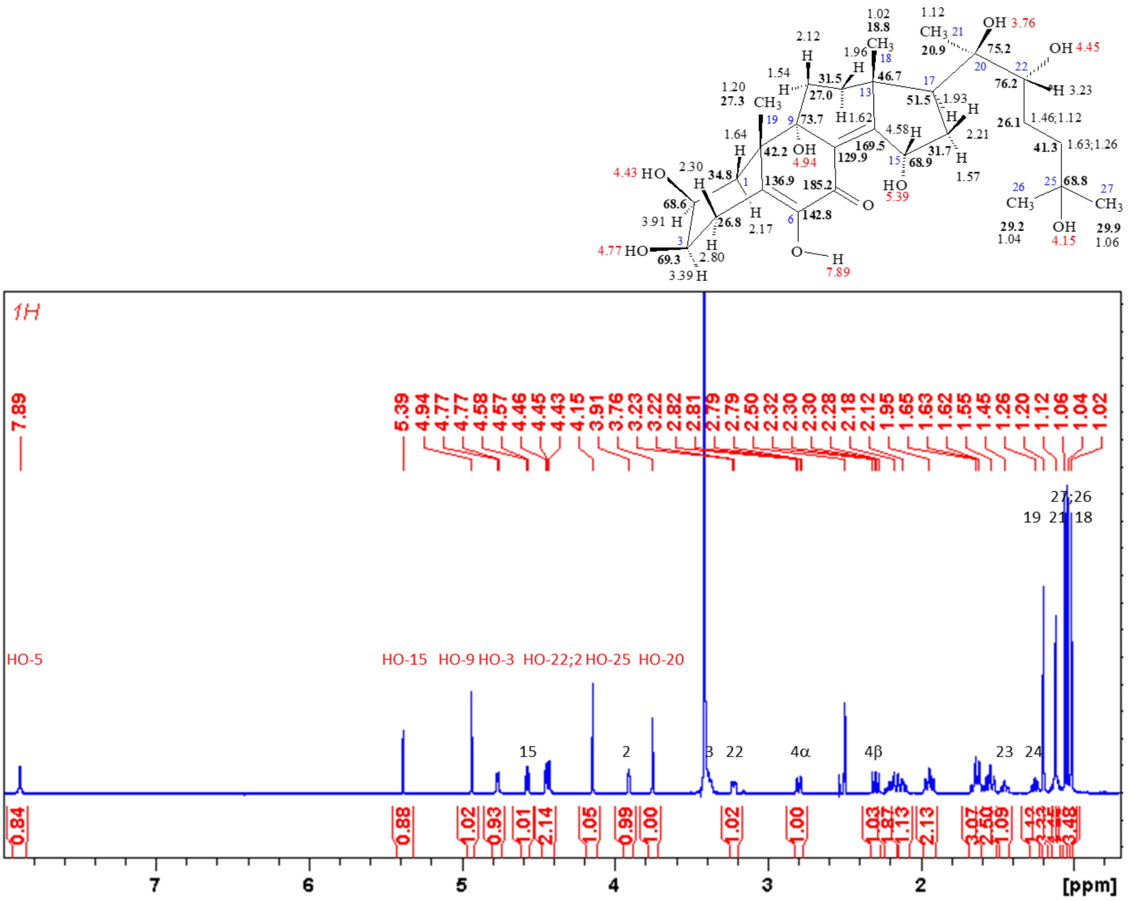

Figure S18. Compound **4**, selTOCSY (τ_{mix}: 80 ms) on H-15, H-2 and H-22

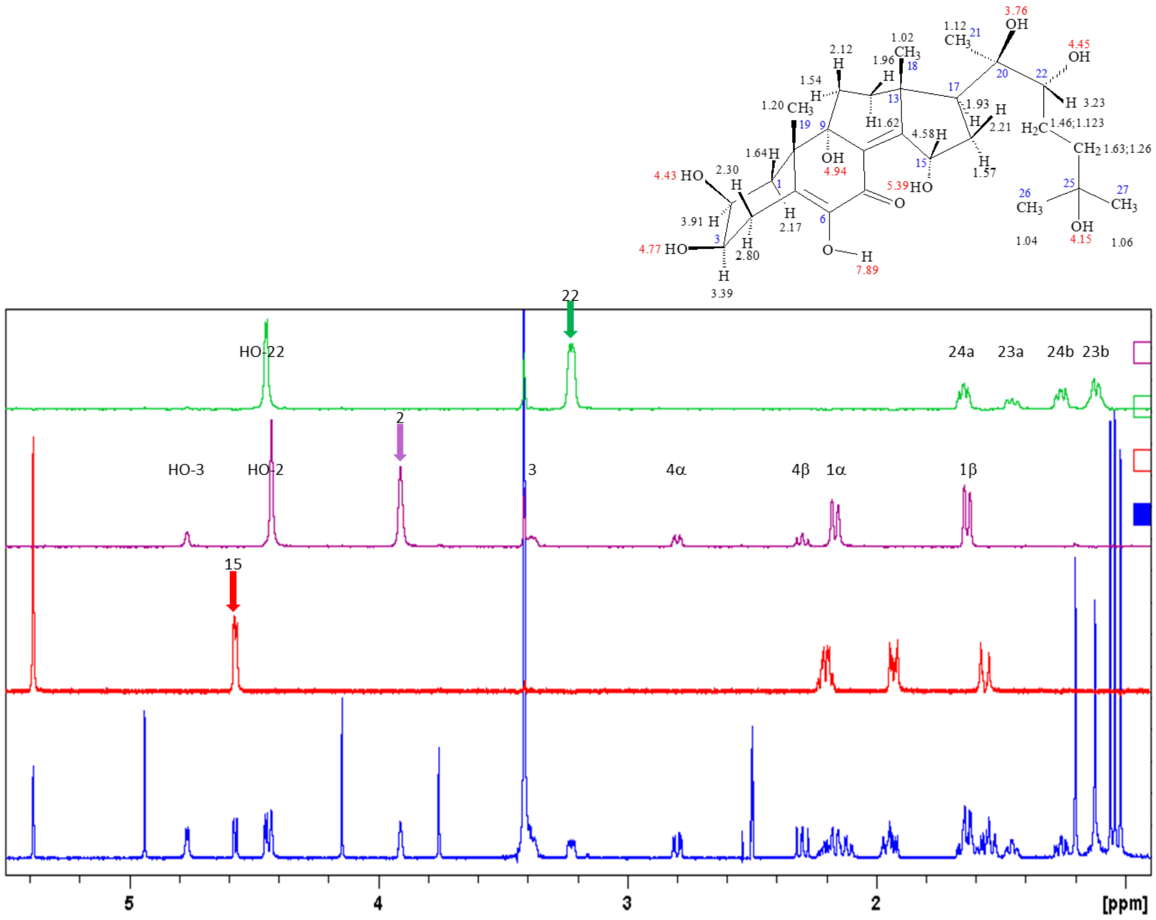

Figure S19. Compound **4**, sel-Roesy (τ_{mix}: 300 ms) on Me-18, Me-21 and Me-19

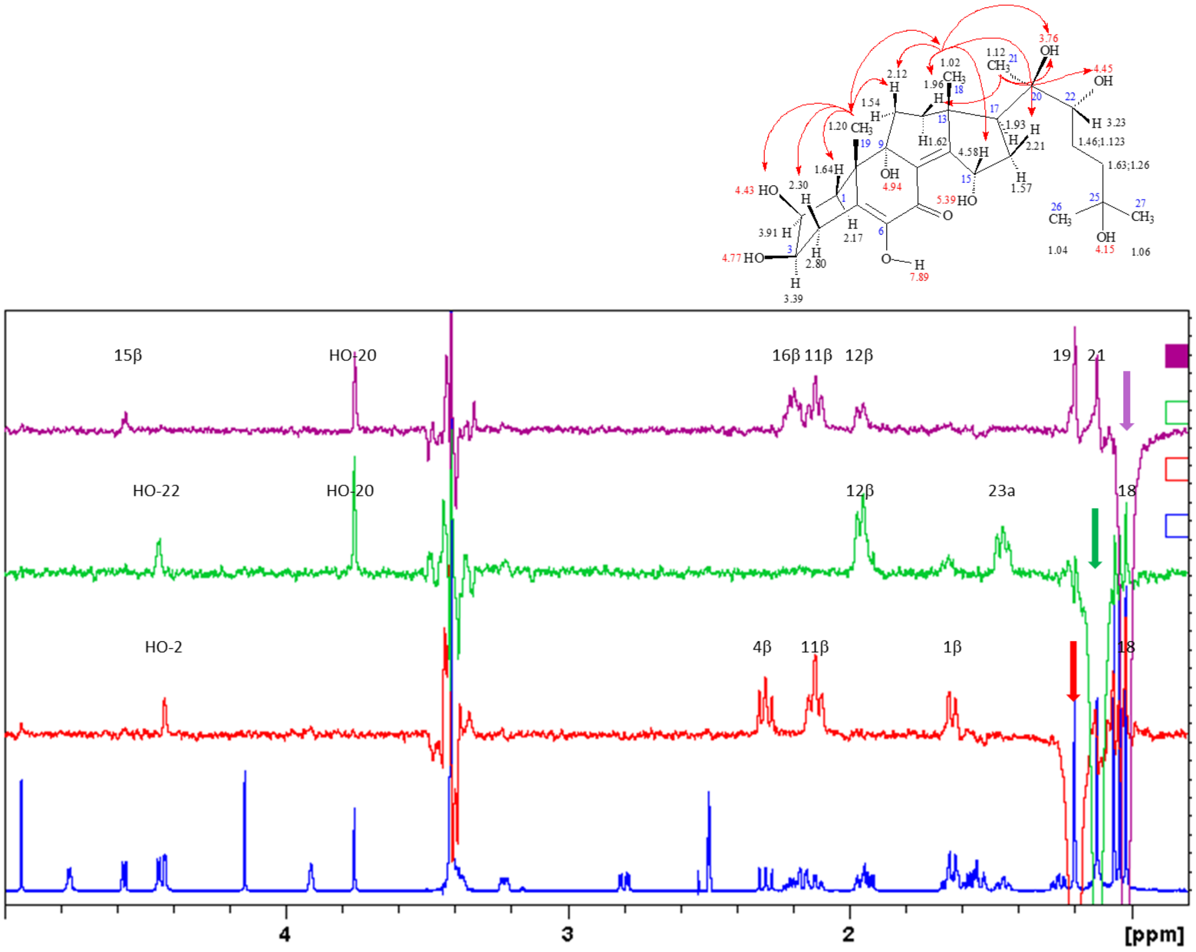

Figure S20. Compound **4**, DEPTQ

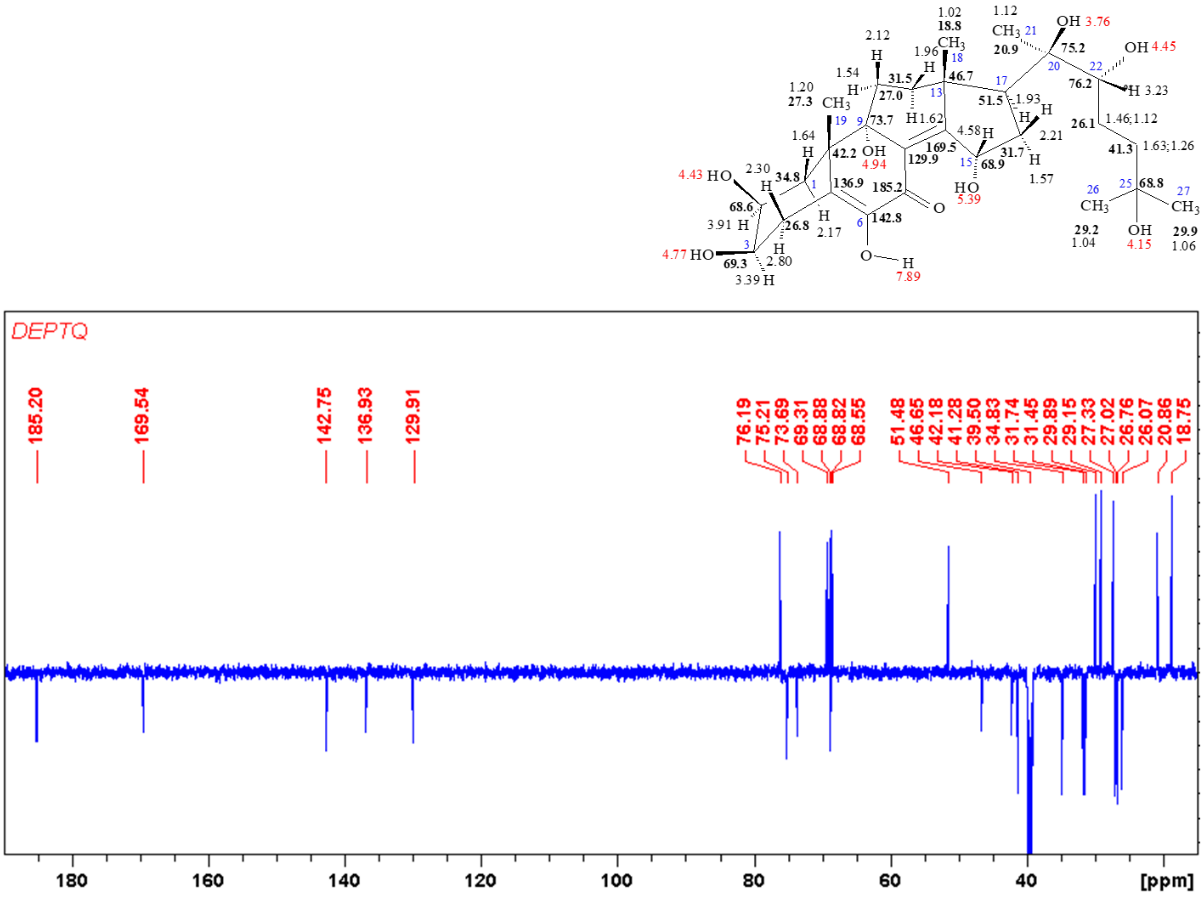

Figure S21. Compound **4**, edHSQC

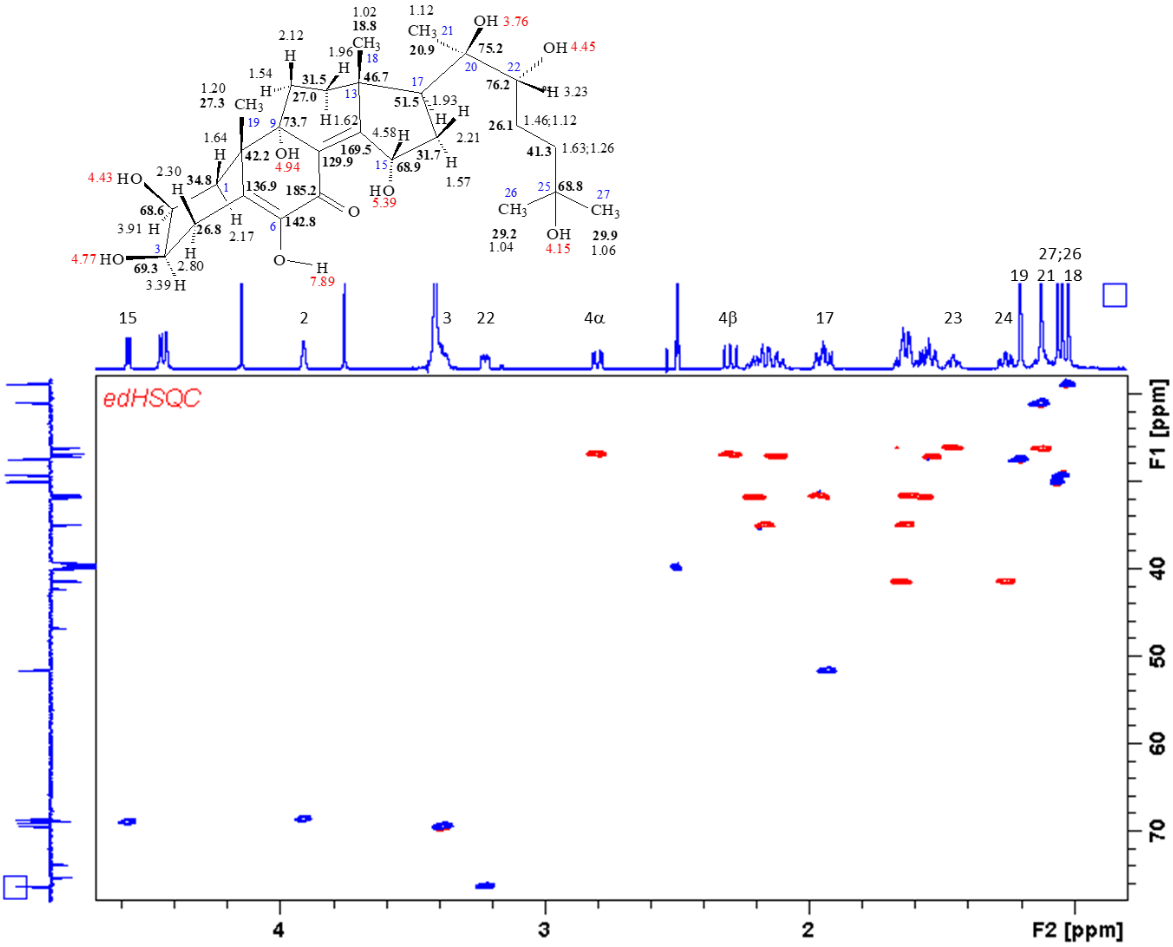

Figure S22. Compound **4**, edHSQC section

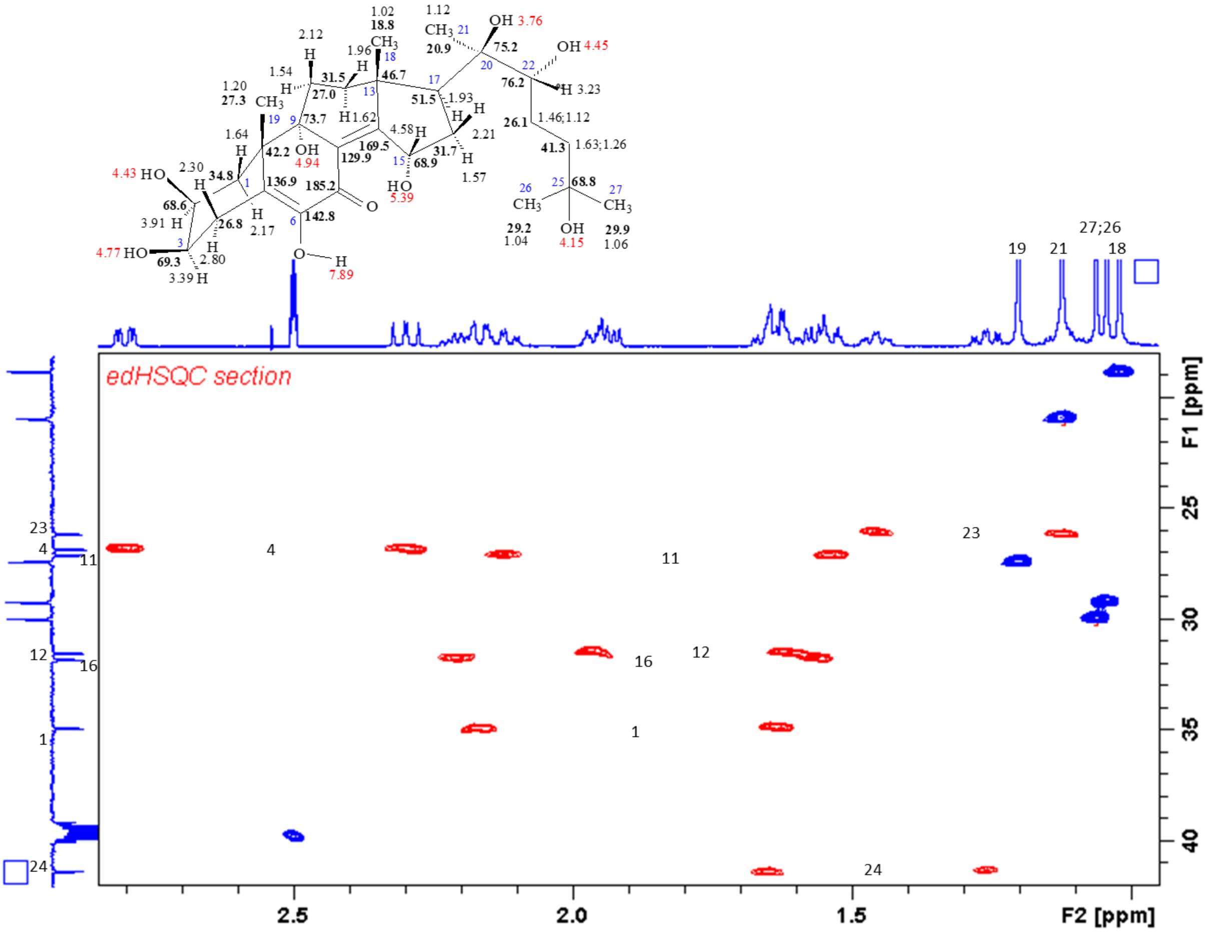

Figure S23. Compound **4**, HMBC and HMBC section

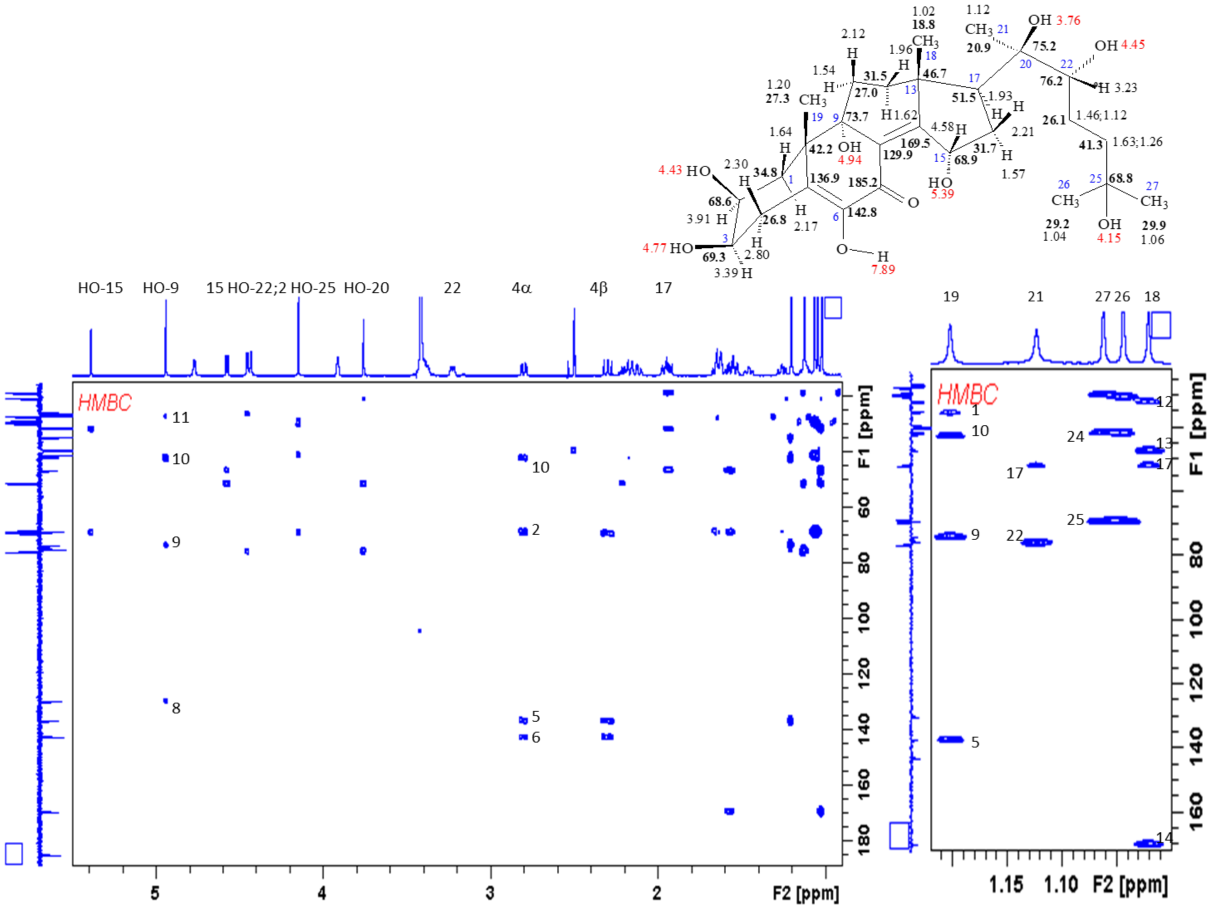

Figure S24. Gradual decomposition of calonysterone (**Cal**) to compound **4** in 50% aqueous methanol.

Column: Kinetex Biphenyl 4.6x250mm, 5 µm; flow: 1 mL/min; PDA detection: λ=200 –550 nm.

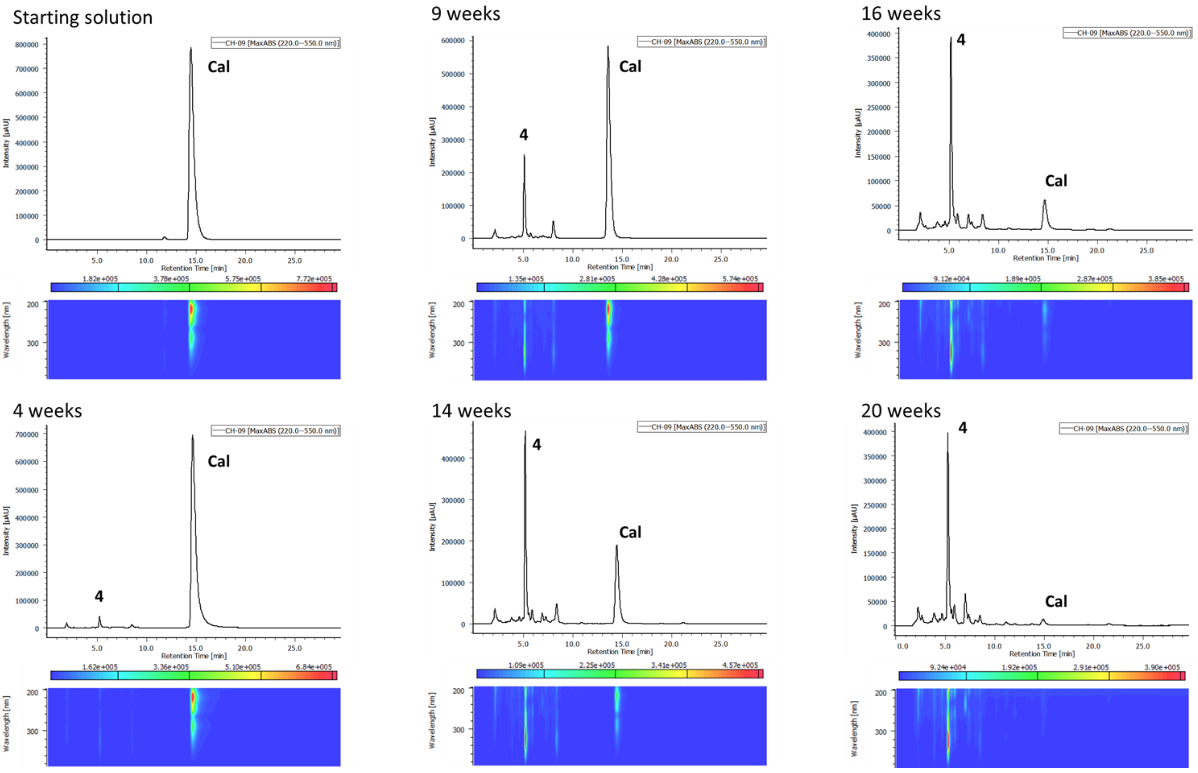

Figure S25. Calibration for the determination of compound **4**.
Calibration line was set to intersect at the origin.

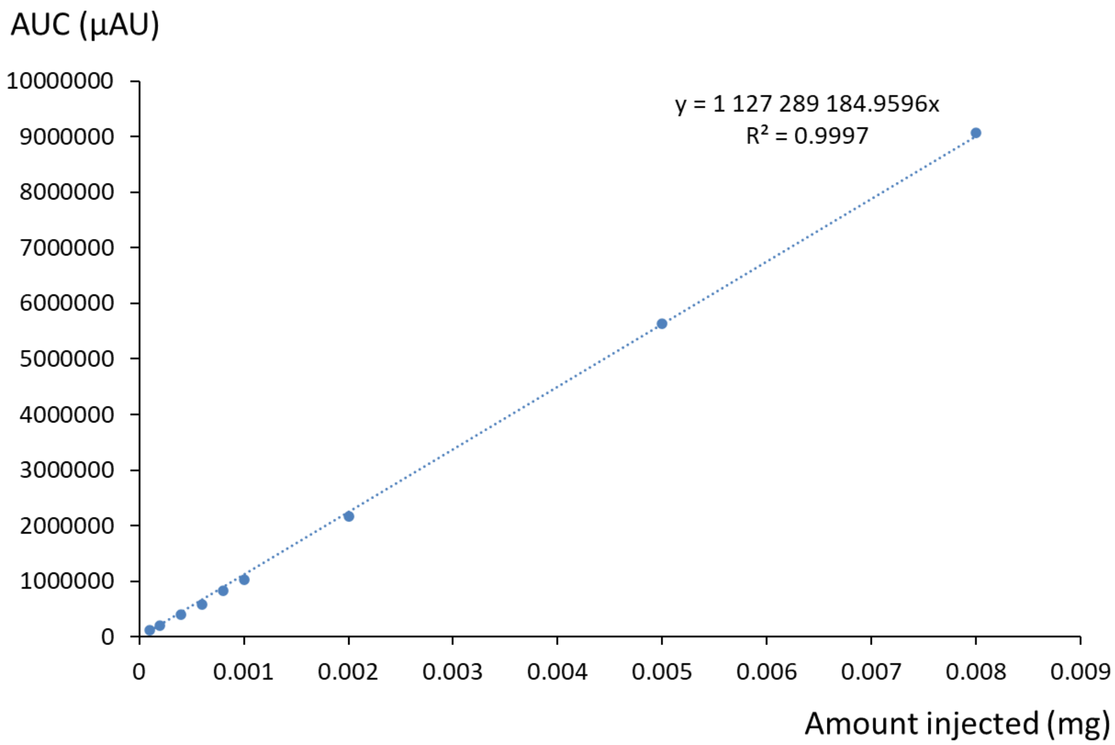

Figure S26. Effect of compounds **2**–**4** on human brain endothelial cell viability

Impedance based cell assays were performed to detect the effects of compounds **2** (**A**), **3** (**B**), and **4** (**C**) on hCMEC/ D3 human brain microvascular endothelial cells (0.01-10 µM concentrations, 4-hours treatment). Values are presented as mean ± SEM, n = 4–25. Data were analyzed by one-way ANOVA followed by Dunnett's multiple comparisons test, ** $p < 0.01$ compared to the control.

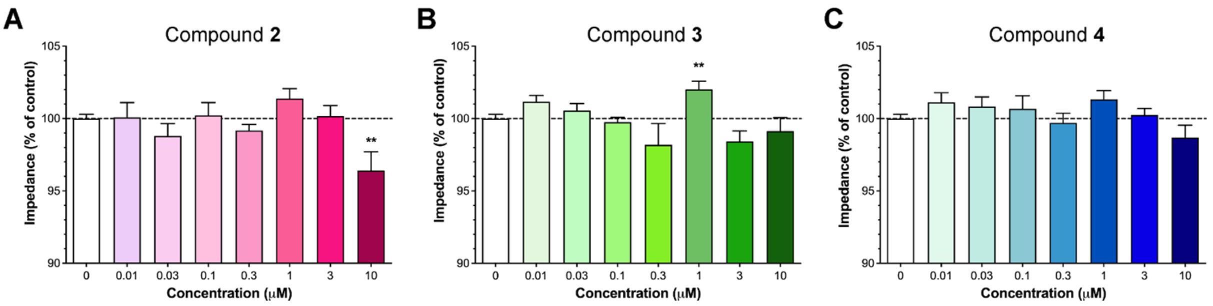

Figure S27. Impedance-based cell viability/barrier integrity assay to detect the effects of α-melanocyte stimulating hormone (α-MSH) on cytokine treated rat brain endothelial cells.

Rat brain endothelial cells were treated with cytokines (10 ng/ml TNF-α and 10 ng/ml IL-1β, 24 h) without or with α-MSH (1 and 10 pM) and the cellular effects were measured by impedance. Control group received culture medium. The cytokines decreased the cell index, which effect could be ameliorated by α-MSH treatment, especially by the lower, 1 pM α-MSH concentration. Means ± S.E.M., n = 3-6, ***P<0.0001, # P<0.05, *: CK, 1 and 10 pM α-MSH+CK compared to C, #: 1 and 10 pM α-MSH+CK compared to CK. C, control group; CK, cytokine treated group.[1]

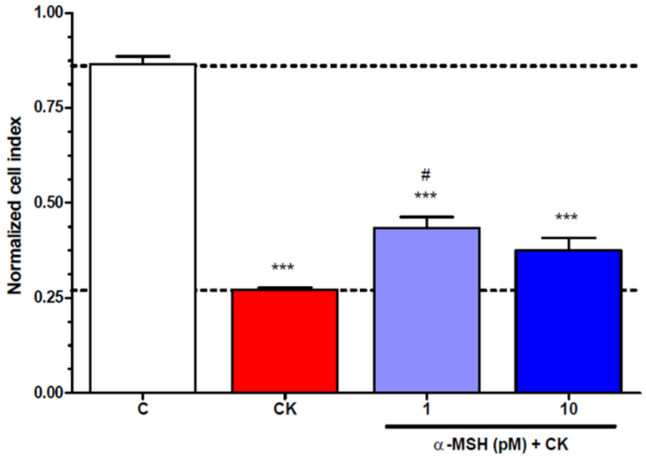

[1] Figure was reproduced from *Harazin et al., PeerJ 2018, 6, e4774*, published under the CC BY 4.0 open access license (ref. 18 in the main manuscript text)
